# Justifications for using complementary and alternative medicine reported by persons with musculoskeletal conditions: A narrative literature synthesis

**DOI:** 10.1371/journal.pone.0200879

**Published:** 2018-07-19

**Authors:** Nadia Corp, Joanne L. Jordan, Peter R. Croft

**Affiliations:** Arthritis Research UK Primary Care Centre, Research Institute for Primary Care and Health Sciences, Keele University, Keele, Staffordshire, United Kingdom; Teesside University, UNITED KINGDOM

## Abstract

**Background:**

Complementary and alternative medicine (CAM) is very popular with patients frequently combining it with orthodox health care. The high prevalence of CAM use and satisfaction with CAM reported by patients directly challenges an orthodox system that can only approve such use if it results from the application of biomedical concepts and science. Studies highlighting this as a cultural, sociological and historical phenomenon emphasise the value of choice for consumers of health care. Musculoskeletal conditions typify common problems for which the effectiveness of orthodox care is often unclear. We postulated that the reasons people give for using or not using CAM for musculoskeletal conditions, would therefore indicate the full range of expectations that people have of health care. Furthermore, these reasons would indicate how much people feel orthodox health care is or is not meeting their expectations. Therefore, this study aims to investigate people’s reasons for choosing or avoiding CAM for non-traumatic musculoskeletal conditions.

**Methods:**

A systematic search and narrative synthesis was conducted of published qualitative and quantitative studies related to CAM and non-traumatic musculoskeletal conditions.

**Results:**

We identified 169 relevant papers detailing 152 separate studies, from which 1486 justifications were extracted concerning CAM use. Content analysis resulted in 11 distinct categories across four themes: practical aspects of care, clinical effectiveness, non-clinical outcomes of care, and a person’s philosophy of illness and care. People provided similar rationales for both using and avoiding CAM, emphasising that, whilst CAM is perceived by many patients with musculoskeletal conditions to fill gaps in care (such as practitioner time or quality of the therapeutic relationship), orthodox care also seeks to deliver these aspects of care. However, people who used CAM also highlighted its alignment with their general philosophy and ideas about illness and health care, and often emphasised CAM’s capacity to give them control over their condition and its treatment.

**Conclusion:**

Currently, CAM appears to have a significant role for patients with common painful long-term conditions in providing choices to enable individual needs to be met.

## Introduction

The model of modern orthodox scientific Western medical practice can be summarised as ‘find the pathological disease underlying a patient’s illness and treat it; identify causal mechanisms underlying the disease and prevent it occurring in the first place’. The sick patient gets better; the incidence of disease declines. Musculoskeletal conditions, in total estimated to be the commonest reason for disability globally [1], contain examples of the application and success of this traditional model—the prevention of rickets, joint replacements for patients with osteoarthritis, and drugs which target and counter inflammation in rheumatoid arthritis.

There is no single model of complementary and alternative medicine (CAM). Some therapies, such as herbal remedies, may be developed, tested and applied entirely within the orthodox biomedical framework. Others, such as traditional acupuncture, have a philosophy and principles of action which do not originate and may not fit with orthodox Western medical science. For many people, this makes much CAM implausible and beyond justification as a rational approach to ill-health.

However, there is now widespread acceptance that Western medicine, for all its successes in diagnosing and treating disease, needs a wider view of the sick person than biology and pathology alone provide and must set care in the wider context of a patient’s personal, emotional, social and cultural life, especially for long-term illness such as common musculoskeletal conditions (back pain, osteoarthritis) for which ‘scientific cures’ are often lacking. This reflects the importance of patient autonomy, self-care, choice and expectations, and growing evidence that wider components of care beyond biomedical treatments can improve response to those treatments [2]. Belief in the potential of a treatment to help, for example, and the shared expectation of patient and clinician in its likely success may have powerful effects in relieving musculoskeletal pain [3].

Attention to this wider context of care has been a feature of the traditional healer in societies across the world, and researchers and CAM proponents have argued that this, and placing support for self-care at the heart of the consultation in many types of CAM, is what CAM can deliver [4,5]. Others have argued that this wider care, although important, should only be built around scientifically proven interventions. Orthodox Western practitioners such as family doctors would also argue that this wider care has long been an important component of conventional health care anyway [6]. Yet CAM is very popular, especially among patients with musculoskeletal conditions [7], and the prevalence of CAM use has been increasing for many years, with patients often combining it with orthodox health care [8,9]. This high volume of use and satisfaction with CAM reported by patients is a direct challenge to a conventional system that can only sanction such use if it results from the application of biomedical concepts and science. It has been studied as a cultural, sociological and historical phenomenon, with conclusions that emphasise the value of choice for consumers of health care [10,11].

Musculoskeletal conditions typify common problems for which the effectiveness of orthodox care is often unclear. We hypothesised that the reasons people give for using or not using CAM for musculoskeletal conditions would highlight the full range of expectations that people have of health care. Furthermore, these reasons would indicate how much people feel orthodox health care is or is not meeting their expectations.

We therefore set out to identify and categorise the body of reasons that people give for seeking out and using CAM for musculoskeletal conditions and the reasons others avoid it, by conducting a systematic search and narrative synthesis of literature related to CAM and musculoskeletal medicine.

## Methods

### Research question

In people with non-traumatic musculoskeletal conditions, what are the justifications given for choosing, continuing, avoiding or discontinuing CAM?

### Inclusion and exclusion criteria

#### Types of studies

Quantitative and qualitative studies of any design were included. Intervention studies were only included if participants were able to choose whether they were allocated to CAM therapy or not and their reasons for choice reported. We did not restrict searches by country or date. However, for practical reasons, all non-English language articles were excluded, as were conference abstracts and studies where the full text could not be retrieved.

#### Types of participants

Individuals of any age, sex or ethnicity with non-traumatic musculoskeletal conditions were included, whether from a general or specific population *e*.*g*. primary care and disease/condition specific population. Studies of post-operative pain, trauma-related conditions *e*.*g*. acute sprains and fractures, and conditions that were primarily neurological *e*.*g*. multiple sclerosis, were excluded if no other non-traumatic musculoskeletal condition was included.

#### Types of interventions

We included any intervention considered a complementary and alternative medicine (CAM), defined as “…‥*health care approaches developed outside of mainstream Western*, *or conventional*, *medicine for specific conditions or overall well-being*.” [12], whether it was being used alongside (complementary) or instead of (alternative) orthodox biomedical healthcare.

CAM covers a large and diverse range of interventions. A list of specific CAM therapies and treatment modalities was devised based on the operational definition identified by the Cochrane Collaboration [13,14], database subject headings, and CAM therapies listed by NHS Evidence, Wikipedia and Natural Therapy Pages [15–17]. This included both practitioner-based care, and self-treatment using over-the-counter products *e*.*g*. homeopathic remedies and herbal preparations. Studies concerning psychotherapeutic interventions were excluded.

To summarise, inclusion criteria for screening were:

- Complementary and alternative medicine (CAM)
- A non-traumatic musculoskeletal condition
- *If* an intervention study, participants were given the choice of whether to use CAM or not
- Justifications for using or not using CAM were explicitly provided by participants
- Original report of an empirical study

### Measures or descriptions of justification

The focus of this review was on any measurement or description concerning individuals’ justifications for use or choice of CAM. This included justifications to begin or continue use of CAM (this may include specific facilitators), and any justifications for not using or discontinuing use of CAM (this may include specific barriers).

### Search method

A comprehensive search strategy was designed to capture as much of the relevant literature as possible. Systematic searches were conducted across six electronic databases (EMBASE, MEDLINE, CINAHL, ASSIA, AMED and Web of Science) from inception to July 2011. The searches were rerun in February 2017 (see ‘Update and assessment of robustness’ section below). The search strategy utilised text word searching in the title or abstract along with the database Subject Headings and combined terms for: i.) General or specific CAM therapies; ii.) General or specific musculoskeletal conditions; and iii.) Justification for CAM use (see S1 Appendix for full search strategy for OVID MEDLINE). For the other databases search terms were adapted to the search capabilities of the database platform.

In addition, key journals not fully indexed in the online databases searched were hand searched (*Social Theory and Health*, *Anthropology and Medicine* and *European Journal of Integrative Medicine*), and reference lists from relevant articles, including all those included in the review, were checked.

### Study selection

The initial screening of papers by title was conducted by one reviewer (NC) by excluding clearly irrelevant articles. At this stage these were primarily articles about non-musculoskeletal conditions *e*.*g*. varicose veins and multiple myeloma, or where the intervention was an orthodox approach *e*.*g*. a specific drug therapy or physiotherapy.

The abstracts of the remaining articles were then assessed independently by two reviewers (NC and JJ) for relevance and were excluded by agreement. The reason for excluding each paper was recorded. In addition to condition and intervention, many non-English language and conference abstracts (where no full text article could be found) were identified for exclusion. If it was unclear as to whether a publication was relevant or not, it was included for the next stage. Full text copies of all remaining papers were then obtained and matched against the inclusion criteria.

#### The process of selecting studies for inclusion or exclusion

All full texts were assessed for inclusion by one reviewer (NC), with the two other reviewers (JJ and PC) independently screening separate samples to check consistency (*n* = 50 and *n* = 24 respectively, representing 11% of the total). There was a high level of agreement between the reviewers (96% and 100% agreement respectively for each of the two ‘second’ reviewers) on which articles to include and exclude. Disagreements were documented and resolved between the reviewers. The number of excluded papers was recorded according to the reason.

### Data extraction

A data extraction form was specifically designed for the review and used to record relevant information from each study in a spreadsheet (see S2 Appendix). Data extraction from each paper involved identifying all distinctive justifications mentioned in that paper, drawing on survey results, qualitative quotes from individuals, and any themes or items extracted by the paper’s authors. One reviewer (NC) conducted the data extraction from all papers and a sample was independently checked by a second reviewer (JJ). The following information was extracted:

- study design
- sample size
- study setting
- country
- age of participants
- percentage of female participants
- medical condition(s)
- CAM therapy/type
- justification for CAM use

### Data synthesis

To comprehensively explore all justifications for CAM use by individuals with non-traumatic musculoskeletal conditions, inclusion of both quantitative and qualitative research was necessary. A narrative synthesis process was used to enable the different forms of evidence to be combined, informed by guidance produced by Popay *et al*. [18].

#### Identification of categories

Content analysis was used to categorise textual data. Using the data entered in the spreadsheet, a list of all the justifications recorded from each paper was produced alongside its unique identifier (*i*.*e*. a separate ‘ID’ number for each recorded justification). Blinded to other details of the study, justifications were coded using a five step approach to identify and categorise the reasons for CAM use or non-use:

- *Step 1*: *Linguistically identical/similar*: justifications which were identically phrased or were identical except for linguistic nuances were identified and coded (with an ‘A’ group code) by NC and reviewed by JJ and PC. For example, “expensive drugs” [ID36], “prescription drugs too expensive” [ID49] and “affordable alternative to expensive prescription drugs” [ID480] were all coded as A36.
- *Step 2*: *Conceptually identical/similar*: justifications which were considered identical/very similar conceptually were identified and coded (with a ‘B’ group code) and provided with a code descriptor by NC and reviewed by JJ and PC. For example, those coded A36 (see above) along with similar justifications coded in Step 1 including “conventional treatment too expensive” [ID961], “cost” [ID372], “low cost compared to medical services” [ID710] and “"*stopped going to physical therapy because couldn’t afford it any more*"” [ID981] were all given the same code (B30 –which can be summarised here as ‘Motive: CAM cheaper than orthodox care’).
- *Step 3*: *Categorisation*: justifications from steps 1 and 2 were sorted and grouped to identify broader categories by NC which were coherent and could be designated with a unifying label. During this process there was a point at which no new categories emerged *i*.*e*. saturation of categories was attained. Preliminary labels were then assigned to each category. For example, those coded B30 (see above) along with similar justifications including those coded as B139 ‘Barrier: CAM perceived/considered too expensive to try’, B6 ‘Discontinued: too expensive, could not afford’, B191 ‘Continue: cost-effectiveness’ and B273 ‘Motive: CAM covered by insurance’ were labelled ‘COST’.
- *Step 4*: Categories identified and labelled in step 3 were presented to the two ‘second’ reviewers. Discussion between all three reviewers led to minor amendments in the contents and labels of some categories e.g. ‘COST’ was merged into the category ‘ACCESS’. Then, using these amended categories, two reviewers (JJ and PC) independently allocated a randomly selected subset of all justifications to the available categories (consisting of 10% of all phrases in each category as allocated by the lead reviewer). This provided a check on the appropriateness and robustness of the categorisations. Disagreements were documented and resolved through discussion between all three reviewers.
- *Step 5*: Finally, all justifications were arranged according to their assigned category and then were checked again independently by all three reviewers to ensure accuracy and consistency of categorisation.

Justifications could be assigned to more than one category: this was particularly pertinent for qualitative studies where multiple reasons were often given in one statement.

### Update and assessment of robustness

The search was rerun at the beginning of February 2017 and new papers fitting the inclusion criteria were identified. Full texts were retrieved and justifications were identified to check if any additional reasons were given beyond those already described. In this way, the robustness of the categorisation was checked, using the criterion that no new justifications would be identified with the publication of new studies.

We followed the PRISMA statement guidelines for reporting systematic review of studies that evaluate health care interventions [19], as far as was relevant for this systematic search and narrative synthesis: see S3 Appendix for PRISMA checklist.

## Results

**Fig 1:**
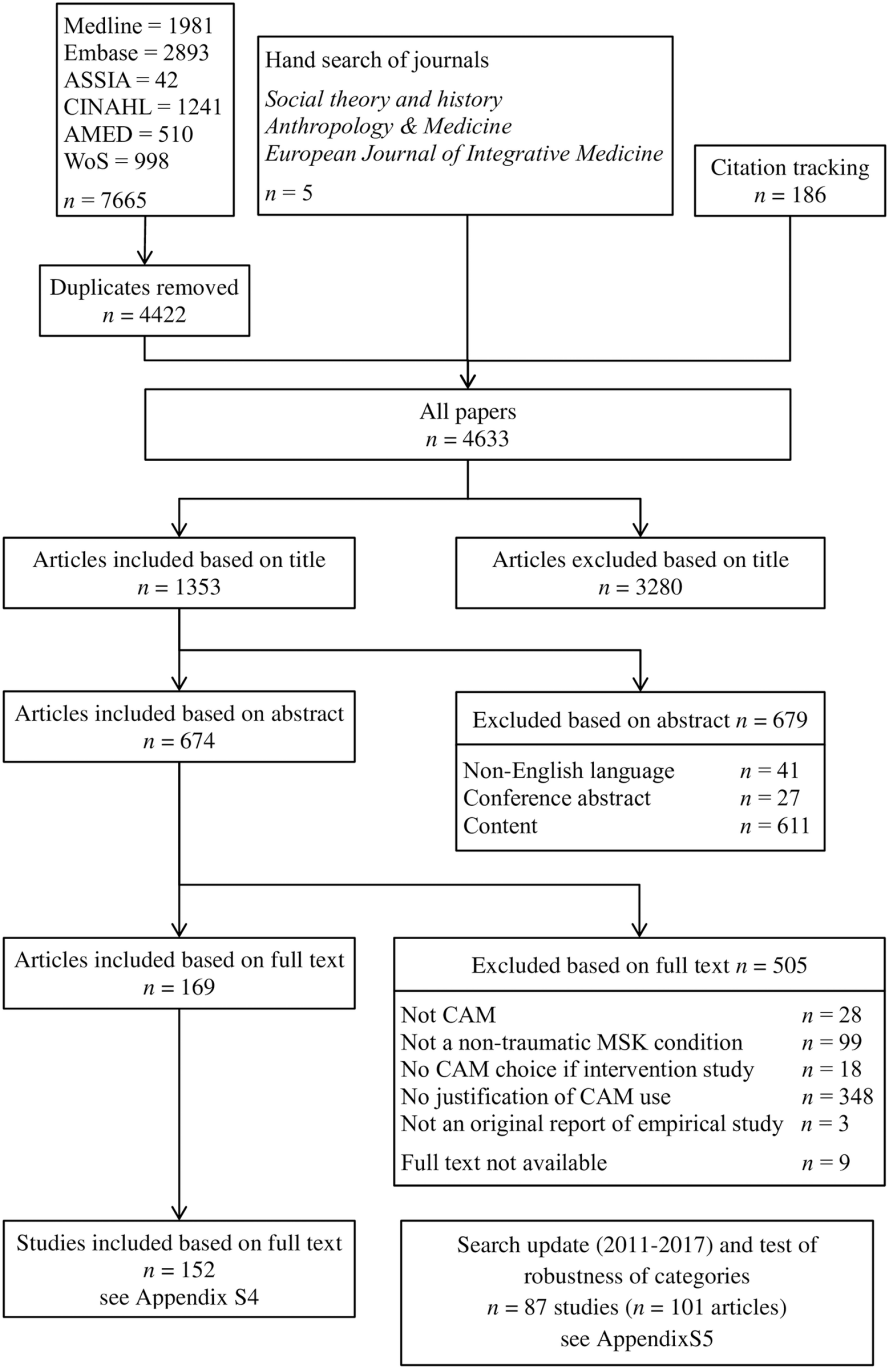
Flow diagram detailing the identification, screening, eligibility and included studies.

One hundred and fifty-two studies, reported in 169 papers (see S4 Appendix for full list of included papers), were identified for inclusion in this narrative synthesis (see Fig 1). Almost three quarters (74.3%) of studies were based in North America (*n* = 67) and Europe (*n* = 46, of which UK = 29); however, Asia (*n* = 16), Australia and New Zealand (*n* = 12), the Middle East (*n* = 9) and Africa (*n* = 2) were also represented.

Orthodox health care was the most frequent setting for studies (*n* = 65, 43%), although population- and community-based settings were also popular (*n* = 49, 32%). In comparison few studies focused on recruiting participants from CAM settings (*n* = 24, 16%), with a minority recruiting from more than one setting (*n* = 14, 9%): the setting was not clear for one study.

There were approximately twice as many quantitative as qualitative studies in population- and community-based or CAM settings, and studies set in orthodox health care were predominantly quantitative (50 vs 10, see Table 1). Sample sizes varied widely (range = 1–44639, see Table 1) but were generally larger in quantitative studies. Nine studies specifically focused on children (*i*.*e*. <18 years old) and one on older people (> = 65 years) but most studies included adults (age = 18–64 years) with children and/or older adults (*n* = 137): five studies did not include the age of their participants.

**Table 1:** Study characteristics presented according to study design.

	Study design		
	Quantitative	Qualitative	Mixed
**Number of studies**	102	38	12
**Setting (*n*)**			
• **Population**	33	14	2
• **Orthodox health care**	50	10	5
• **CAM setting**	15	7	2
• **Mixed/miscellaneous**	4	6	3
• **N/K**		1	
**Sample size (*n*)**			
• **Mean n ± s.d.**	1224 ± 5449.3	37 ± 49.2	114 ± 157.1
• **Median (interquartile range)**	152 (92.25–386.75)	26 (13.75–54.5)	58.5 (37.75–105.5)
• **Minimum**	19	1	17
• **Maximum**	44639	221	5754
**Female (%)**			
• **Median (interquartile range)**	66.45 (54.38–76.90)	77.80 (62.07–100)	73.55 (61.10–80.00)
• **Minimum**	11.7	36.8	50
• **Maximum**	100	100	90.3
• **Missing data**	12	1	2
**Musculoskeletal only (*n*)**			
• **Yes**	33	28	6
• **No**	68	9	6
• **Missing data**	1	1	0

A total of 1486 justifications concerning CAM use were extracted across all papers. These embraced reasons concerned with the use of CAM, barriers to using CAM, and the continuation or discontinuation of CAM. Justifications often related to influences from orthodox medicine, both negative *e*.*g*. perceived ineffectiveness of orthodox treatment driving CAM use, and positive, *e*.*g*. a particular CAM therapy being suggested by a doctor.

Eleven distinct categories were identified regarding individuals’ reasons for seeking CAM interventions, namely:

- accessibility and convenience
- confidence
- control of healthcare
- desperation
- effect
- goal
- presentation of condition
- referral
- safety
- satisfaction with health care
- therapeutic environment

They are listed here alphabetically and no particular priority was assumed. However, in the section below, they have been grouped under four thematic sub-headings for purposes of discussion. We also included an additional miscellaneous category for justifications outside the designated eleven. All categories are outlined and discussed below with examples.

The diversity of papers in terms of demographics, study design, health conditions and CAM therapies precluded the identification of patterns between these parameters and the different categories of justification for use or avoidance of CAM.

### Theme A. Practical aspects of care

#### Access and convenience

Key terms associated with this category: Access, convenience, cost, time, practicality and availability.

The category of access and convenience encompasses factors influencing physical access to CAM or orthodox care including cost, availability and time, and also the perceived convenience and practicality of using CAM or orthodox treatment. Rationale and barriers to CAM use were equally in evidence in this category, stressing that this is to do with practical aspects of choice.

Cost of treatment was frequently cited both as a reason to use or continue use of CAM, often when CAM was a cheaper alternative to orthodox care or covered by insurance or an employer *e*.*g*. “*affordable alternative to expensive prescription drugs”* [20], and conversely as a barrier or reason to discontinue CAM use particularly if it was the more expensive option or an additional cost *e*.*g*. “*unable to afford it* [CAM]” [21], or not covered by insurance *e*.*g*. “*lack of insurance coverage for CAM therapies”* [22]. The potential of cost to have a significant influence on choice of CAM was exemplified eloquently by this participant in one study: “*…so I was scared of the chiropractor and of course I couldn’t afford it either*, *so there was massage therapy—I couldn’t afford that either*. *Which one is cheapest*? *Acupuncture*! *So I looked through yellow pages and there was one and I said I’ll give him a call”* [23].

Where CAM was available locally, the ease with which individuals could access it relative to accessing orthodox health care could be a motivating factor, as shown in this example: “*physicians delivering or prescribing alternative therapies more accessible… than physicians prescribing biomedical options”* [24]. Not surprisingly if CAM was not available in the vicinity this became a barrier to CAM use *e*.*g*. “*lack of local native healers”* [25].

Access issues specifically relating to time influenced the use of CAM, namely “*regular appointments*” [26] and the push away from orthodox medicine due to waiting times *e*.*g*. “*waiting for a long time for consultation in hospital or clinics is unpleasant”* [27]. The other mention of time was “*lack of time*” [28], proffered as a barrier or reason to discontinue CAM use.

Convenience and practicality also influenced CAM use. On occasion the reason for CAM use was simply cited as “*convenient access*” [29]; more often however, it was the inconvenience associated with CAM that was a reason to discontinue its use—“*inconvenient to prepare herbal medicine*” [30] or *“inconvenient to visit*” [30] and “*less convenient than Western medicine”* [31]. On a more practical level, CAM could be “*in forms easy to use*” [32], although this was not always the case and then orthodox medicine was seen as the more practical option *“I don’t think I want to take the time with it…*.*[I prefer] going in and popping a pill*” [33].

#### Referral

Key terms associated with this category: advised, suggested, referred, knowledge, information, supplied/bought, curiosity, experimentation and previous experience (not specifically regarding effectiveness).

Referral, both formally and informally, to CAM therapy was frequently identified in the data. This broad category encompasses not only formal referral to, or prescription of, CAM by healthcare professionals *e*.*g*. GP, but also advice, suggestions and recommendations of trusted others, the provision of CAM by family or friends and on occasion doctor, information provided via the media, curiosity and experimentation, and finally awareness.

Three major sources of referral were prominent: general (family) practitioners and other healthcare professionals; family and friends; and the media including TV, radio, magazines, books and the internet. But also, to a lesser extent testimonials from trusted others including patients with a similar condition, co-workers and famous individuals.

For many individuals it appears that their use of CAM was predicated on their doctor’s formal referral, suggestion or approval *e*.*g*. “*…GP who told me first that Harpagophytum was relevant for arthritis*.*”* [24] and “*I never heard of it until the doctor told me*.*”* [28]. Conversely CAM’s non-use was often associated with lack of referral to CAM and the doctor’s disapproval *e*.*g*. “*I’m not using nothing the doctor hadn’t prescribed for me*.*”* [34] and “*doctor would disapprove”* [35].

The other main barriers were a lack of awareness or information about CAM and lack of referral by non-medical others, *e*.*g*. “*lack of information about where to obtain treatments or whether treatments would be useful”* [22] and “*If I had somebody who could tell me*, *you know*, *with a testimonial or whatever*, *and they got rid of it…*.” [28].

#### Desperation

Key terms associated with this category: Despair, helplessness, hopelessness, last resort, nothing else worked and try anything that might work.

Desperation became a motivation for seeking CAM therapy when all other routes had been exhausted and CAM was seen as the final place of hope *i*.*e*. patient’s ‘last resort’, as articulated in the following quotes: “*no other solution for their problem”* [36], “*it’s that feeling of hopelessness; I’m willing to go past my comfort…*.*outside my belief system…*.*use something that may be unproven”* [37], “*well*, *it was a last resort—I was feeling a lot of pain and hoped radon would help*. *When you hurt you’ll try anything”* [20] and “*open to trying anything that might work for the pain”* [21].

### Theme B. Clinical effectiveness of care

#### Effect

Key terms associated with this category: effective; ineffective; effect; help; work; balance; success; benefit; previous experience of effectiveness and to supplement/add benefit.

The perceived effectiveness of treatments was one of the more frequent categories identified within the data. Motivations for CAM use in this category were broadly divided into those for which CAM was considered an effective approach, or more effective approach than orthodox treatment, in generally managing or treating a condition or symptoms *e*.*g*. “*effective in treating RA* [Rheumatoid arthritis]” [30], “*I’ve seen it do wonders on other people that have either gone through cancer or chemotherapy or whatever*” [23] and “*I remember my grandmother putting some sliced pickled cucumber on my hands…*.*cured it*. *So I tried that again*, *and it’s actually pretty good*” [38] and “*Chinese medicines are more effective than Western medicines because they are capable of building strength”* [39]; and those where orthodox treatments were found ineffective *e*.*g*. *“prescription medicine not working”* [40], *“failure of conventional treatments to relieve symptoms*” [41] and *“I was taking that* [orthodox medicines] *and the pain came back*, *so I tried moxa cautery”* [38].

There was frequent mention of CAM providing additional benefit, usually in relation to concurrent orthodox treatment *e*.*g*. “*both Western and Eastern forms of medical care can complement each other nicely*, *one supplying the patient with treatments the other may be lacking*. *This can only help all patients stay as well as possible*.*”* [42].

Furthermore, perceived effectiveness was one of the major factors determining the continuation or discontinuation of a CAM therapy *e*.*g*. “*participants sustained the decision to use alternative therapy by evaluating whether* “balance in mind-body-spirit” *or* “balance between the inner and outer person” *had been achieved”* [37] and “*I was doing those capsules for yonks* [glucosamine/cod liver oil supplements], *like*. *Because those things are long term anyway*, *and I didn’t feel any benefits from it to be quite honest*” [43].

The barriers to CAM use associated with this category were: the view that CAM was not effective *e*.*g*. *“homeopaths are not effective”* [44] or was less effective than orthodox medicine “*some patients reported that herbal supplements were not as effective* [as Western medicines]” [45], or had too mild and/or too slow an effect *e*.*g*. “*treatment is long and results are slow to appear*” [44]. In contrast to negative aspects of CAM precluding their use, another reason for CAM non-use was the success of orthodox medicine *i*.*e*. “*pharmacologic therapy already working”* [28].

#### Goals

Key terms associated with this category: improve, alleviate, relieve, enhance, increase, cure and control of condition or symptoms.

In some cases, the reason for using CAM was to achieve a specific goal *i*.*e*. a specific beneficial outcome, often in relation to a particular symptom or condition as the following examples highlight: “*long-term relief of symptoms”* [46], “*help relieve pain better”* [47], “*to increase energy”* [48], “*improve their general health”* [49], “*relief of my fibromyalgia*” [50], and “*about once a month I tell her I need a limberin’ up job”* [51]. Others used CAM preventatively *e*.*g*. *“to prevent disability*” [52] or “*to prevent disease progression*” [53], or had a more mechanistic reason for using CAM *e*.*g*. “*supposed to get in there and work them joints and loosen ‘em up”* [34] or “*to replenish nutrients”* [54].

No perceptions of orthodox medicine were stated as underlying any reason within this category for using CAM. However, orthodox medicine was referred to when used in conjunction with CAM, with each having specific goals *e*.*g*. “*In treating a disease like mine*, *Western medicine was my first choice*, *I chose Chinese medicine for my recovery and for health promotion”* [also Control] [55].

#### Confidence

Key terms associated with this category: Confidence, credibility, legitimacy, trust, faith, expectation/belief, evidence, proof, training and expertise.

An individual’s confidence in treatments or practitioners also figured in both reasons for and against CAM use. In some cases, it was simply expressed as trusting or having faith in a particular CAM therapy *e*.*g*. “*I believe in it”* [8]; others were more pragmatic and their motivation was underpinned by the need for what they considered credible treatments with an evidence base *e*.*g*. “…*there has even been a write up in the Lancet about it and there is real proof now that it really is helpful*‥*”* [43] and “…*I think there’s quite a lot more evidence to support osteopathy than some or the other alternative”* [29], or suitably qualified practitioners *e*.*g*. “*knowing the people in charge would be properly qualified and the students properly trained*" [29] and “*belief that an excellent Chinese healer can bring about miracles by using secret therapies that have been passed down generation to generation or created by themselves”* [56]. On occasion CAM use was also driven by negative perceptions of orthodox medicine, predominantly a “*lack of trust in medical treatment*” [57].

Barriers to using CAM therapies reflected the antithesis of the reasons for use: lack of belief in CAM *e*.*g*. “*not into quack remedies like yin and yang”* [58] or lack of credibility *e*.*g*. “*never been to a chiropractor*, *I’ve always been a bit suspicious that they’re slightly quackery*, *I don’t really know…*.*since the medical profession haven’t endorsed them as much as they’ve endorsed physiotherapists I’ve tended to trust the judgement of the medical profession*" [58] or reputable practitioners “*I would actually like to look at alternative therapies*, *complementary therapies*, *I guess the difficulty is finding a practitioner who had a reputation*‥*”* [also ‘Access’] [43]. One study highlighted that *“most patients were concerned they might be laughed at”* [59] if they used CAM.

### Theme C. Other outcomes of care

#### Satisfaction

Key terms associated with this category: satisfaction, happy, disappointment, disillusioned and frustration.

The category of satisfaction was dominated by dissatisfaction, disappointment and frustration with orthodox healthcare or healthcare professionals, driving individuals to use CAM, as typified by “*doctors are hopeless”* [46], “*dissatisfaction with conventional health care*” [60] and “*feeling bad about usual treatment e*.*g*. “it’s just pills and pills and pills and pills”” [61]. In contrast, reference to satisfaction with CAM was given as a motivating factor *e*.*g*. “*satisfaction with this type of therapy*” [62] or “*CAM was superior*, *was better in terms of quality of health care and services”* [63].

Barriers to CAM included dissatisfaction with CAM, but also satisfaction with current orthodox care *e*.*g*. *“satisfied with care and not considered an alternative”* [64].

#### Safety

Key terms associated with this category: side-effects, contraindications, interactions, adverse effects, safety and allergy.

Safety issues formed another category of motives for the use of CAM. CAM therapies were often considered to be safe, with no or few side effects. “*a comparative lack of unpleasant side effects”* [65] “*alternative therapies are less harmful to my body than prescription drugs*” [66] and “*Complementary medicine does not have side effects”* [67]. The side effects of orthodox medications were a prominent driver in the use of CAM, whether actually experienced or a concern, or indeed to see if CAM could ameliorate the side effects experienced with allopathic drugs “*some experimented by taking western medicines and herbal supplements together to see if the herbs would counteract the side effects of their tablets”* [45]. Similarly, perceived side effects provided barriers to CAM use “*Lord*, *no*! *That’d eat the skin off your body”* [34] and “*I don’t want anything to interfere with what I am already taking*. *Counteracting or whatever”* [68] and actual experience of side effects led to discontinued use “*adverse effects including stomach discomfort or skin irritation*” [69] and “*it caused me problems or side effects”* [8].

### Theme D. Philosophy of illness and care

#### Presentation

Key terms associated with this category: severity, chronicity, ill-defined symptoms, suitability of treatment and type of conditions/problem.

On occasion, the general presentation of the condition was found to have a bearing on the use of CAM. On the one hand CAM was favoured for chronic and/or serious conditions *e*.*g*. “*chronic illness should be treated by a sinseh”* [39] and “*I have a serious illness with poor chance of recovery*” [70], or where the condition occurred in a younger person or quickly progressed *e*.*g*. “*having a child with a chronic condition”* [71] and “*when progression was rapid and/or occurred at a relatively young age the disease generated greater concern and people tended to seek treatment wherever it might be available”* [72]. On the other hand, non-serious conditions with mild or vague symptoms also elicited CAM use *e*.*g*. “*ill-defined or mild symptoms*” [65], often in conjunction with the perception that the condition did not warrant orthodox treatment *e*.*g*. “*problem was not serious enough for their physician”* [73], or that orthodox treatment was not the most suitable intervention for their symptoms or condition *e*.*g*. *“…I’ve got more of a feeling that my body is not functioning properly*. *You can’t go to your GP for this…”* [65], “*Some participants chose HPs* [health professionals] *on the basis of the perceived type of pain e*.*g*. *chiropractic for nerve pain or massage therapy for muscular pain”* [58] and “*I cannot take prescription drugs because of my health condition”* [66].

#### Therapeutic environment

Key terms associated with this category: natural, therapeutic relationship, consultation time, therapeutic approach, philosophy/belief system, holistic, therapeutic physical environment and support/understanding.

The therapeutic environment was often identified in studies as driving the use of CAM. This encompassed the physical environment of CAM, or orthodox treatments, the therapeutic encounter and the nature and underlying philosophies of CAM. Several references were made to CAM being ‘natural’, ‘holistic’, ‘individualised’ and ‘able to address the underlying cause’, often in contrast to orthodox medicine. Examples include “*I prefer the natural solution to the problem*” [52], “*I value the emphasis on treating the whole person*” [74], “*CAM considers the interrelatedness of mind*, *body and spirit*” [21], “*more enjoyable*, *more holistic*, *gentler and more individualized than conventional treatments”* [65], “*more personal attention to their patients than conventional practitioners*” [41], "*a focus on identifying causes or* “the root” *of a problem* " [65], “*using these types of remedies and treatments is consistent with my beliefs*” [75] and “*Complementary medicine* ‘made sense’” [76].

The therapeutic encounter was also an important aspect, specifically consultation times and therapeutic relationships between the therapist and patient, not only drawing people to CAM, *e*.*g*. “*participating in clinical decisions”* [77] and “*a more equal relationship with my complementary practitioner than with my doctor”* [74], but conversely driving people away from orthodox medicine, *e*.*g*. medical doctors “‥*did not understand their problem*” [78], or were “‥*not interested in their case”* [78], “‥*did not give me enough time*” [74] or “*found it difficult to talk to my doctor”* [74]. There was also recognition that “*allopathic physicians* “don’t have all the answers”” [37].

Only a few barriers to CAM use were identified in this category: some mentioned their religious belief as precluding CAM use, another raised the issue of CAM practitioners being unable to diagnose.

#### Control of healthcare

Key terms associated with this category: choice, preference, avoid and proactive.

Often there was mention of individuals taking control and being proactive with regard to their own healthcare, as shown in the following comments “*reluctant to put up with it* [pain]. *I wanted to do something”* [79], “*I believe that complementary therapy enables me to take a more active part in maintaining my health”* [74] and “*psychologically*, *I feel as though I am actually doing something*. *I feel in control*” [80]. But this also could mean being in control and having the choice of which practitioner was seen, or which treatment was received: “*being able to select their own practitioner*” [77] and “*personal preference*” [81].

Reactions to orthodox medicine as a driver for CAM use were also identified in this category, and were primarily associated with limiting or avoiding orthodox interventions, for example, “*to avoid long term drug taking”* [24], the “*desire for symptomatic relief without the use of allopathic medication*” [82], “*fear or avoidance of surgery*” [32] and “*I want to stay with my own knee*, *no matter what*” [52].

### Theme E. Miscellaneous

#### Miscellaneous

Only four reasons fell into the miscellaneous category. One regarding CAM use “*became users because of coincidence*” [78]; the remaining 3 were barriers to CAM use, including “*no interest”* [73], lack of opportunity *e*.*g*. “*not having a reason to try”* [78], and “*not wanting to be seen as a complainer*” [58].

### Robustness

A total of 87 new studies, reported in 101 articles, published between July 2011 and the beginning of February 2017 were pertinent to this review (see S5 for full list of papers included in robustness testing). The full texts were scrutinised and justifications for CAM use/non-use were cross-referenced against the reasons identified in the original search. No additional reasons were identified, thus supporting the robustness of the categorisation presented above.

## Discussion

We have identified quantitative and qualitative empirical work that describes and reports on the reasons why people use or do not use CAM for the treatment of common musculoskeletal pain syndromes. This reflects a substantial literature, and does not rely only on samples drawn from people surveyed or interviewed in CAM settings.

CAM is not one single entity or monolithic practice; it embraces many different settings and types of care, with a highly variable ‘fit’ with Western notions of mechanism and evidence of effectiveness. This heterogeneity, however, is also true of orthodox medicine. We accept that lumping together many different practices under the heading of ‘CAM’ and ‘orthodox medicine’, and treating them as separate entities, is artificial. However, there is a general public perception of CAM that reflects its definition as “*a group of diverse medical and health care interventions*, *practices*, *products or disciplines that are generally not considered part of conventional medicine*” [12]; and in this paper the term ‘orthodox medicine’ covers what people consider to be part of conventional medicine. The large number of studies that ask people about their perceptions of CAM and orthodox medicine suggests this is a practical framework for enquiry and analysis, despite the boundaries between the two being “*grey and mobile and culturally bound*” [12].

Our review has not addressed the evidence base for effectiveness of either CAM or orthodox medicine in the treatment and care of patients with musculoskeletal conditions, nor entered the debate about the status and appropriateness of different forms of evidence in judging usefulness or importance to health care and health policy [83]. Our starting point was the high prevalence of CAM use for musculoskeletal conditions. Such high prevalence must, we argue, represent important patient expectations about health care perceived as being relevant to their condition and which patients judge as being met by CAM or not met by orthodox medicine. Our paper reviewed the reasons why people do or do not use CAM as one component of understanding and describing these expectations. Whether such information is seen as relevant to improving orthodox care, justifying CAM, or contributing to a pragmatic pluralist and integrated model of care for patients with chronic pain and disability from musculoskeletal conditions [84], is for debate elsewhere.

We identified eleven distinct justification categories, grouped into four themes. The first theme was practicalities of care. As Porter observed of 17^th^ Century London, choice has always been rated highly by patients seeking symptom relief [85]; 21^st^ Century medicine now embraces this in tackling issues like travel distance and waiting times. Lay and professional recommendation or referral provide practical authority to choose treatments when there is no obvious and immediate cure, reflecting the importance of public culture in driving CAM use and the frustration of orthodox practitioners treating musculoskeletal conditions.

Perceived effectiveness was a second theme, regardless whether this was backed by orthodox scientific evidence or not. People experiment for themselves. This is discomforting and problematic for Western scientific thought, because the individual’s rationale is based on a belief that CAM helps; but that belief may rest on claims that ignore Western notions of science and evidence. CAM also meets more general expectations of effectiveness and provides confidence, but so also does orthodox medicine. Most patients with musculoskeletal conditions in Western countries use both forms of health care [8], working out for themselves what each provides.

The third theme identified outcomes other than clinical effectiveness. Satisfaction with care and safety of care are now considered important outcomes for orthodox health care also [86]. Dissatisfaction with orthodox care was a common justification for using CAM, as was the perception of orthodox medicine as risky and CAM as safe. However, CAM was not consistently viewed as safe, which was why some discontinued or did not use CAM. Given the variable efficacy and risk profile of common treatments for pain such as non-steroidal anti-inflammatory drugs, it is hardly surprising that a sceptical public turns to things that appear to be safe.

The fourth theme finds CAM meeting a need among patients confronted by the medicine of high technology and scientific interventions—namely for care that coheres with broader aspects of life and motivates self-help. As Braunack-Mayer and Avery observed about why people go to the doctor “*the logic that they (the patients) follow in deciding to seek help may not resonate with clinicians*, *since it will be shaped by a wider range of factors than the straightforwardly biomedical*” [87]. CAM of course does not have exclusive hold on these justifications, since many health care professionals provide the same thing, or would do given time and inclination. People make choices about who they think may understand and help them—even presenting the same illness complaints in different words to orthodox and CAM practitioners [88]. Our review simply highlights that some patients with musculoskeletal problems see CAM is good at meeting these needs for time, support for self-management, and holistic care.

### Comparison papers

General reviews of perspectives on CAM and general population surveys have highlighted comparable categories to those identified here. Bishop et al.’s review of beliefs predicting CAM use identified a sense of control and participation, beliefs about holism and natural treatments, and general philosophies of life [89]. One US survey found CAM use emphasised its congruency with users’ own values, beliefs, and philosophical orientations toward health and life [90]; another that users of both CAM and orthodox care regarded the combination as superior and expressed similar confidence in each component [91].

There have been studies in other disease groups, such as patients with cancer, and groups expected to be more ‘resistant’ to the attractions of CAM, which confirm the generalisability of the justifications emerging here. Shumay et al. found that patients with cancer who used CAM wished to avoid damage or harm to the body, and some reported an unsatisfactory relationship with health care providers [92]. The authors concluded that orthodox care had potential to ensure these needs (patient education, improvements in physician-patient communication, and psychologic therapy) were met through combining conventional and CAM treatments.

The traditional medical model, according to Soler and Okkes, provides an incomplete framework for primary care generally: “*there is more to life than medicine may diagnose*, *and family medicine should strive to move closer to the lives of our patients than the medical model alone could allow*” [93]. Paskins et al., reviewed studies about why patients consult their general practitioner about osteoarthritis, and found health professionals’ negative attitudes were a disincentive, paralleling our findings that some people report that CAM provides a more positive therapeutic environment [94].

### Strengths and limitations

Our aim in this review was to summarise empirical evidence about the reasons given by patients with musculoskeletal conditions for using or avoiding CAM.

The choice of a broad inclusion category of all musculoskeletal conditions was based on important features of these conditions. They are by far the commonest group of conditions globally that cause continuing disability but low mortality over time [1] and pose challenges for patients and health care professionals as how best to manage the symptoms of persistent pain and reduced activity in daily life, with often accompanying symptoms of depression and anxiety [95]. They are now the commonest of long-term pain conditions in people with other chronic diseases including cancer survivors [96]. There is strong evidence that persons with these conditions such as osteoarthritis and back pain are the highest users of CAM in the general population and that they often use CAM and orthodox treatments in combination [8]. Studies of people with these conditions were highly likely to deliver a comprehensive range of justifications for use of CAM in a field (chronic pain) where there is less controversy about the plausibility of CAM use than in treating highly specific disease pathologies. For this reason, we also excluded traumatic MSK conditions because they represent (often as acute diagnosed pathologies) a different set of problems to those presented by common daily pain in conditions of the back, neck, muscle and joints.

We chose to exclude psychotherapy treatments and practitioners from our search (for example, private counsellors, and mindfulness practitioners). In our analysis and discussion of a number of the justification categories, it is clear that one of the attractions of CAM for many people is the perceived promotion of mental well-being achieved through both the therapeutic relationship, and the time, context and style provided by a number of CAM therapies. Orthodox health care also aims to provide this in routine health care, but more specifically through counselling and psychotherapies. Therefore, this was one area where the overlap between orthodox and CAM practice is likely to be difficult to disentangle and so for the particular purposes of this study, these were excluded.

Strengths include the comprehensiveness and inclusiveness of the literature search, and the blinded independent assessment of categories that emerged for classifying the wealth of reasons which appear in the literature. On the other hand, the inclusion of quantitative as well as qualitative studies was a potential limitation, given that ‘closed’ options may restrict or influence respondents’ choice as compared with qualitative material, and the quotes we have used to illustrate categories are more likely to reflect qualitative sources.

Another potential limitation of our study is that its methods are not those of a conventional systematic review and lie in the middle of the spectrum of review ‘types’. However, we would argue that this is a strength in relation to the aim and task of the work described in this paper. We wanted to undertake a systematic search of all relevant peer-reviewed English language literature in order to supply the fullest possible range of sources of published ‘justifications’ for CAM use and non-use, and hence provide an extensive and comprehensive pool (or ‘denominator’) of justifications for our analysis. This was not a search only for studies whose principal aim was to investigate justifications, so quality assessment or selection by study method was inappropriate. Whilst we recognise the potential that justifications in some papers might be selective, we are reassured by the fact that many justifications came from studies in non-CAM settings and that our updated 2017 search indicated that saturation of commonly expressed justifications for both use and non-use of CAM is likely to have been reached.

The second limitation that arises from the particular choice of methods is that a structured narrative was adopted to follow the systematic search for all published justifications. However, this is a likely strength in relation to the aim of the analysis—namely, to use robust methods (grouping by independent observers and cross-validation) to classify justifications as the basis for discussion without any selective assumptions of what are ‘valid’ justifications. The whole point of the paper is to present an accessible summary and account of all justifications for use and non-use as the basis for debate about the needs of patients that are or are not being met by both CAM and orthodox medicine.

Lastly, a further limitation was that non-English language literature was excluded for practical reasons of scale; it may be that further categories or a different balance of justifications for CAM use would emerge if other languages had been included. However, our systematic search identified studies from many countries across the globe, making it less likely that culture-specific justifications were missed.

## Conclusions

Whether rational evidence-based clinicians focused on the biomedical model of disease like it or not, many people like CAM, and their justifications align with criticisms of the shortfall of Western medicine in providing a fully effective and satisfying system of health care. In the current climate of evidence-based medicine underpinning national recommendations for care, it is unlikely that the commissioning of CAM by health services will reflect its popularity. A fruitful way forward for health care systems may be to use the justifications that people have for using CAM as a framework to address the shortfalls and problems of their own systems. The extent to which CAM itself should be included in national policy and clinical practice to address those gaps in health care remains a topic for debate, including about the nature of evidence that should underpin choice of care for long-term conditions such as chronic musculoskeletal pain [97].

## Supporting information

S1 Appendix OVID MEDLINE search strategy. (DOCX)

S2 Appendix Data extraction Form. (DOCX)

S3 Appendix PRISMA checklist. (DOC)

S4 Appendix All articles included in the narrative synthesis. (DOCX)

S5 Appendix All articles included in robustness testing. (DOCX)
