# Supplementary material for: Justifications for using complementary and alternative medicine reported by persons with musculoskeletal conditions: A narrative literature synthesis
Source: PLoS One. 2018 Jul 19;13(7):e0200879. doi: 10.1371/journal.pone.0200879 (PMC6053199; doi:10.1371/journal.pone.0200879)
Supplement: S1 Appendix — (DOCX) [file pone.0200879.s001.docx]

**Appendix 1: OVID MEDLINE search strategy**

The following table is an explanation of the symbols used in the search strategy below.

/ indicates an index term (MeSH heading)

exp before an index term indicates that all subheading were selected

ad after an index term indicates administration & dosage

th after an index term indicates therapy

tu after an index term indicates therapeutic use

.ti,ab. indicates a search for a term in title/abstract

* at the end of a term indicates that this term has been truncated.

adj indicates a search for two terms where they appear adjacent to each another

adj*n* indicates a search for two terms where they appear within *n* words of each another

1 attitude/

2 exp Attitude to Health/

3 Choice Behavior/

4 Decision Making/

5 patient preference/

6 attitud*.ti,ab.

7 percept*.ti,ab.

8 expectation*.ti,ab.

9 experienc*.ti,ab.

10 preferen*.ti,ab.

11 choice*.ti,ab.

12 belie*.ti,ab.

13 opinion*.ti,ab.

14 priorit*.ti,ab.

15 benefi*.ti,ab.

16 reason*.ti,ab.

17 decision*.ti,ab.

18 motiv*.ti,ab.

19 justif*.ti,ab.

20 (concern or concerns or concerned).ti,ab.

21 (view or views or viewed).ti,ab.

22 exp Consumer Satisfaction/

23 patient satisfaction/

24 satisf*.ti,ab.

25 or/1-24

26 exp pain/

27 pain.ti,ab.

28 26 or 27

29 exp back/ or back.ti,ab.

30 knee/ or knee.ti,ab.

31 shoulder/ or shoulder.ti,ab.

32 Neck/ or neck.ti,ab.

33 elbow/ or elbow.ti,ab.

34 exp hand/ or hand.ti,ab.

35 wrist.ti,ab.

36 hip/ or hip.ti,ab.

37 exp foot/ or (foot or feet).ti,ab.

38 ankle.ti,ab.

39 or/29-38

40 28 and 39

41 (((whiplash adj associated) or whiplash-associated) adj disorder*).ti,ab.

42 (chronic adj3 pain).ti,ab.

43 (pain adj syndrome*).ti,ab.

44 (reflex adj sympathetic adj dystrophy).ti,ab.

45 fibromyalgia.ti,ab.

46 exp Musculoskeletal Diseases/

47 Rheumatology/

48 musculoskeletal.ti,ab.

49 rheumat*.ti,ab.

50 (joint* adj3 disease*).ti,ab.

51 arthriti*.ti,ab.

52 arthrosis.ti,ab.

53 arthralgia*.ti,ab.

54 osteoarthr*.ti,ab.

55 coxarthr*.ti,ab.

56 gonarthr*.ti,ab.

57 ankylosis.ti,ab.

58 (sjogren's adj syndrome).ti,ab.

59 osteoporosis.ti,ab.

60 (intervertebral adj disk adj (degeneration or displace*)).ti,ab.

61 (spinal adj stenosis).ti,ab.

62 spondylarthr*.ti,ab.

63 spondylitis.ti,ab.

64 spondylosis.ti,ab.

65 osteochondritis.ti,ab.

66 (plantar adj fasciitis).ti,ab.

67 (heel adj spur).ti,ab.

68 metatarsalgia.ti,ab.

69 (((temporomandibular adj joint) or TMJ) adj disorder*).ti,ab.

70 gout*.ti,ab.

71 pseudogout*.ti,ab.

72 bursitis.ti,ab.

73 (impingement adj syndrome).ti,ab.

74 (dupuytren* adj contracture).ti,ab.

75 (muscular adj dystroph*).ti,ab.

76 (polymyalgia adj rheumatica).ti,ab.

77 tendinopath*.ti,ab.

78 tenosynovitis.ti,ab.

79 epicondylitis.ti,ab.

80 (tennis adj elbow).ti,ab.

81 Carpal Tunnel Syndrome/

82 (carpal adj tunnel).ti,ab.

83 myositis.ti,ab.

84 myopath*.ti,ab.

85 myalgia.ti,ab.

86 dermatomyositis.ti,ab.

87 polymyositis.ti,ab.

88 hypermobility.ti,ab.

89 (fatigue adj syndrome*).ti,ab.

90 osteonecrosis.ti,ab.

91 osteomalacia.ti,ab.

92 (paget's adj disease).ti,ab.

93 exp Vasculitis/

94 vasculiti*.ti,ab.

95 (arteriti* or polyarteriti*).ti,ab.

96 exp Scleroderma, Systemic/

97 Scleroderma, Localized/

98 scleroderma.ti,ab.

99 exp Lupus Erythematosus, Cutaneous/

100 exp Lupus Erythematosus, Systemic/

101 lupus.ti,ab.

102 or/40-101

103 exp Complementary Therapies/

104 integrative medicine/

105 CAM.ti,ab.

106 (complementary adj2 (medicin* or therap* or treatment*)).ti,ab.

107 (complimentary adj2 (medicin* or therap* or treatment*)).ti,ab.

108 (alternative adj2 (medicin* or therap* or treatment*)).ti,ab.

109 (unconventional adj2 (medicin* or therap* or treatment*)).ti,ab.

110 (integrat* adj (medicin* or therap* or treatment*)).ti,ab.

111 (holistic adj (therap* or treatment* or medicin*)).ti,ab.

112 (Anthroposophical adj (therap* or treatment* or medicin*)).ti,ab.

113 Ayurved*.ti,ab.

114 (traditional adj3 (medicin* or heal*)).ti,ab.

115 (chinese adj medicin*).ti,ab.

116 (unani or yunani).ti,ab.

117 (folk adj medicin*).ti,ab.

118 kampo.ti,ab.

119 shaman*.ti,ab.

120 sidda.ti,ab.

121 (naturopath* or nauropath*).ti,ab.

122 homeopath*.ti,ab.

123 (energy adj1 (medicin* or heal*)).ti,ab.

124 exp Magnetic Field Therapy/

125 ((electromagnet* or magnet*) adj2 therap*).ti,ab.

126 (electrotherap* or (electric adj2 therap*)).ti,ab.

127 (phototherap* or (light adj therap*)).ti,ab.

128 (colourpuncture or colorpuncture).ti,ab.

129 (chromotherap* or ((colour or color) adj therap*)).ti,ab.

130 (crystal* adj (therap* or treatment* or heal*)).ti,ab.

131 Laser Therapy, Low-Level/

132 laser/ad, th, tu

133 (laser* adj2 (therap* or treatment*)).ti,ab.

134 reiki.ti,ab.

135 (therapeutic adj touch).ti,ab.

136 (healing adj touch).ti,ab.

137 (distan* adj heal*).ti,ab.

138 (SHEN adj therap*).ti,ab.

139 Kairos.ti,ab.

140 (polarity adj (treatment* or therap* or heal*)).ti,ab.

141 (brennan adj heal*).ti,ab.

142 bioenergetic*.ti,ab.

143 biofeedback.ti,ab.

144 neurofeedback.ti,ab.

145 (jin adj shin adj jyutsu).ti,ab.

146 (qigong or (qi adj (gong or kung))).ti,ab.

147 (chigong or chikung or (chi adj (gong or kung))).ti,ab.

148 (spiritual adj heal*).ti,ab.

149 (theta adj2 (heal* or therap*)).ti,ab.

150 (faith adj heal*).ti,ab.

151 (prayer* or praying).ti,ab.

152 witchcraft.ti,ab.

153 magic.ti,ab.

154 (body adj (re-alignment or realignment or alignment)).ti,ab.

155 (soul adj (re-alignment or realignment or alignment)).ti,ab.

156 (life adj (re-alignment or realignment or alignment)).ti,ab.

157 (reconnective adj (heal* or therap*)).ti,ab.

158 (transcenden* adj (heal* or therap*)).ti,ab.

159 (mind-body adj medicin*).ti,ab.

160 acupuncture/

161 acupuncture.ti,ab.

162 acupressure.ti,ab.

163 (acustimulation or (acupoint adj stimulation)).ti,ab.

164 (trigger adj point*).ti,ab.

165 electroacupuncture.ti,ab.

166 auriculotherap*.ti,ab.

167 moxibustion.ti,ab.

168 shiatsu.ti,ab.

169 relaxation.ti,ab.

170 meditation.ti,ab.

171 ((deep-breathing or (deep adj breathing)) adj exercis*).ti,ab.

172 (hypnosis or hypnotherap*).ti,ab.

173 ((visualisation or visualization) adj technique*).ti,ab.

174 (guided adj imagery).ti,ab.

175 yoga.ti,ab.

176 ((tai adj chi) or (t'ai adj chi) or "tai ji" or taiji).ti,ab.

177 Manipulation, Spinal/

178 ((manipulative or manipulation) adj (therap* or treatment*)).ti,ab.

179 ((body-based or (body adj based)) adj therap*).ti,ab.

180 (craniosacral or (cranial adj sacral)).ti,ab.

181 (spinal adj manipulation).ti,ab.

182 kinesiolog*.ti,ab.

183 Osteopathic Medicine/

184 osteopathic physicians/

185 osteopath*.ti,ab.

186 chiropractic/

187 chiropract*.ti,ab.

188 (tui adj na).ti,ab.

189 reflexolog*.ti,ab.

190 cupping.ti,ab.

191 scraping.ti,ab.

192 coining.ti,ab.

193 spooning.ti,ab.

194 (movement adj therap*).ti,ab.

195 pilates.ti,ab.

196 (alexander adj (method* or technique*)).ti,ab.

197 (rosen adj method*).ti,ab.

198 gyrotonic*.ti,ab.

199 feldenkreis.ti,ab.

200 naprapath*.ti,ab.

201 (bowenwork or (bowen adj (therap* or technique or work))).ti,ab.

202 (zero adj balancing).ti,ab.

203 (bodywork or (body adj work)).ti,ab.

204 (body adj harmony).ti,ab.

205 (aston adj (patterning or kinetics)).ti,ab.

206 (soft adj tissue adj (manipulation* or mobilisation* or mobilization*)).ti,ab.

207 (neuromuscular adj facilitation).ti,ab.

208 (neuromuscular adj therap*).ti,ab.

209 (neuromuscular adj retraining).ti,ab.

210 (muscle adj energy adj technique*).ti,ab.

211 (metamorph* adj (therap* or method* or treatment* or technique*)).ti,ab.

212 rolfing.ti,ab.

213 trager.ti,ab.

214 hellerwork.ti,ab.

215 (structural adj integration).ti,ab.

216 (functional adj integration).ti,ab.

217 (postur* adj alignment adj therap*).ti,ab.

218 (neuroskeletal adj (re-alignment or realignment or alignment)).ti,ab.

219 massage.ti,ab.

220 (amma adj therap*).ti,ab.

221 Bindegewebsmassage.ti,ab.

222 (caring adj touch).ti,ab.

223 effleurage.ti,ab.

224 (lymph* adj drainage).ti,ab.

225 (dorn adj2 (method* or therap* or treatment*)).ti,ab.

226 exp plants, medicinal/ad, tu, th

227 exp plant extracts/ad, tu, th

228 exp plant oils/ad, tu, th

229 oils, volatile/ad, tu, th

230 ((plant* or herb*) adj1 (medicin* or therap* or treatment* or remed*)).ti,ab.

231 phytotherap*.ti,ab.

232 herbalis*.ti,ab.

233 ((flower or tree) adj essenc*).ti,ab.

234 ((flower or tree) adj remed*).ti,ab.

235 (bach adj2 remed*).ti,ab.

236 exp Diet Therapy/

237 functional food/

238 health food/

239 vegetarian*.ti,ab.

240 macrobiotic*.ti,ab.

241 ((functional or health) adj food*).ti,ab.

242 exp Dietary supplements/ad, tu, th

243 (diet* adj supplement*).ti,ab.

244 exp Vitamins/ad, tu, th

245 exp Minerals/ad, tu, th

246 exp Fish Oils/ad, tu, th

247 exp Dietary Fats, Unsaturated/ad, tu, th

248 exp Tissue Extracts/tu

249 (shark adj cartilage).ti,ab.

250 apitherapy/

251 exp Bee Venoms/ad, tu, th

252 bees/

253 honey/

254 honey.ti,ab.

255 propolis/

256 propolis.ti,ab.

257 pollen.ti,ab.

258 (royal adj jelly).ti,ab.

259 (bee* adj (sting* or venom)).ti,ab.

260 ((pet or animal) adj2 (therap* or treatment*)).ti,ab.

261 (chelation adj therap*).ti,ab.

262 (speleotherap* or halotherap*).ti,ab.

263 Transcutaneous Electric Nerve Stimulation/

264 (transcutaneous adj2 (nerve adj stimulation)).ti,ab.

265 exp balneology/

266 balneotherap*.ti,ab.

267 (hydrotherap* or aquatherap*).ti,ab.

268 (bath or baths or bathing).ti,ab.

269 (mud adj (therap* or treatment*)).ti,ab.

270 (floatation or float or floating or floatworks).ti,ab.

271 Sensory Deprivation/

272 (restricted adj environmental adj stimulation adj (therap* or treatment*)).ti,ab.

273 thalassotherap*.ti,ab.

274 ammotherap*.ti,ab.

275 (sound adj therap*).ti,ab.

276 (art adj therap*).ti,ab.

277 (music adj therap*).ti,ab.

278 (dance adj therap*).ti,ab.

279 (play adj therap*).ti,ab.

280 (laughter adj therap*).ti,ab.

281 (aromatherap* or (aroma adj therap*)).ti,ab.

282 ((hopi or ear) adj candl*).ti,ab.

283 Radiesthesia.ti,ab.

284 (Hyperbaric adj oxygen* adj therap*).ti,ab.

285 (ozone adj therap*).ti,ab.

286 prolotherap*.ti,ab.

287 ((colonic or colon) adj (irrigation or therap*)).ti,ab.

288 or/103-287

289 interview/

290 exp Interviews as Topic/

291 interview*.ti,ab.

292 (focus adj group*).ti,ab.

293 exp Tape Recording/

294 (audiorecording or (audio adj recording)).ti,ab.

295 (taperecording or (tape adj recording)).ti,ab.

296 (videorecording or (video adj recording)).ti,ab.

297 exp Questionnaires/

298 questionnaire*.ti,ab.

299 population surveillance/

300 health care surveys/

301 qualitative*.ti,ab.

302 survey*.ti,ab.

303 narration/

304 narrat*.ti,ab.

305 Observation/

306 observation*.ti,ab.

307 (poll or polls).ti,ab.

308 ((thematic or content) adj analys*).ti,ab.

309 or/289-308

310 25 and 102 and 288 and 309
