# Supplementary material for: Justifications for using complementary and alternative medicine reported by persons with musculoskeletal conditions: A narrative literature synthesis
Source: PLoS One. 2018 Jul 19;13(7):e0200879. doi: 10.1371/journal.pone.0200879 (PMC6053199; doi:10.1371/journal.pone.0200879)
Supplement: S2 Appendix — (DOCX) [file pone.0200879.s002.docx]

**Appendix 2: Data extraction form**

**Justification For The Use Of Complementary And Alternative Therapies In Treating Musculoskeletal Conditions: A Systematic Review**

**DATA EXTRACTION FORM**

**Reviewer**       **Date**

**Paper / Ref**       **Publication year**

**Inclusion/exclusion of paper**

Include? Yes  No

Reason for exclusion:

**Study details:** tick all that apply

Systematic review

Cohort study

Cross-sectional survey

Qualitative research

Random sample

Purposive sample

Representative sample

Convenience sample

Interview

Focus group

Questionnaire

Multiple choice  Open  Mixed (open & MC)  Unsure/not clear

Telephone

Face-to-Face

Postal

Other, please specify

**Survey (if applicable):**

Response rate:

**Population:**

| No. of people in sample: |  |
| --- | --- |
| Study setting (general practice, CAM therapy, general population, rheumatology outpatients etc) | |
| Sampling/recruitment process: | |
| Study dates (if known): | |

***Population Characteristics:*** *please include all statistics available including ranges, means, medians, any categorisation etc.*

**Does the study ONLY include children** yes No

| City, Country |  |
| --- | --- |
| Age |  |
| Sex |  |
| Education |  |
| Ethnicity |  |
| Employment status |  |
| Socio-economic group and/or income |  |
| Marital status |  |
| Medical insurance |  |
| Other relevant information/grouping |  |

**Medical condition(s) included in study** (tick all that apply)**:**

**Does the study ONLY include MSK disorders/diseases:** yes No

| MSK/Rheumatology, generally | Fibromyalgia | Pain, Lower back |
| --- | --- | --- |
| Arthritis (unspecified) | Osteoporosis | Pain, MSK (specific, detail below) |
| Rheumatoid arthritis | Non-MSK (specify below) | Pain, Chronic (unspecified) |
| Osteoarthritis | Pain, MSK (unspecified) | Pain, Chronic MSK (detail below) |
| Gout | Pain, Back | Headache |
| **Other conditions, please specify (all):** | | |

| **Number and/or percentage of total with condition:** include all relevant statistics |
| --- |

| **Duration of health condition/problem** |
| --- |
| **Time since diagnosis specifically** |

**CAM therapy or therapies –** please use exact description used in the paper

| ***Named Specific therapies:*** | | |
| --- | --- | --- |
| Acupressure | Massage | Shiatsu |
| Acupuncture | Mediation | Spiritual Healing |
| Alexander Technique | Minerals | Supplements |
| Aromatherapy | Naturopathy | Tai Chi |
| Aromatherapy massage | Omega (3,6, and/or 9) | Therapeutic Touch |
| Chiropractic | Osteopathy | Traditional Chinese Medicine |
| Fish oils | Qigong | Vitamins inc. multi- & mega- |
| Herbal Medicine/Herbalism | Reflexology | Yoga |
| Homeopathy | Reiki |  |
| Hydrotherapy | Relaxation |  |
| ***Broader categories indicated:*** | | |
| CAM – unspecified | Complementary therapy  unspecified | Alternative therapy  unspecified |
| Biologically-based therapy | Dietary/Nutritional | Manipulative therapies |
| Body-based therapy | Energy therapy | Manual therapy |
| Holistic therapy | Folk remedies | Mind-body therapy |
| **Other therapies or categories included:** please specify giving as much detail as possible. | | |

| **Grouping of therapies:** please detail any groupings made in the study and which therapies fall into which grouping within the study |
| --- |

**Use of CAM therapies**

| **Number and/or Percentage of sample using each therapy:** include any relevant statistics |
| --- |
| **Number of CAM therapies used by individuals:** include any relevant statistics |
| **Duration of CAM treatments** *i.e.* nos of treatment sessions or length of time therapy received over |

| **Satisfaction with CAM treatments:** include any relevant statistics |
| --- |

| **Justification for use of CAM therapy:** *include any influences e.g. suggestion of friends, GP recommendation, media information etc., and give any related statistics* |
| --- |

| **Other information pertinent or of potential interest:** |
| --- |

| **Author’s conclusion:** |
| --- |

| **Reviewer’s comments:** |
| --- |
