# Supplementary material for: Justifications for using complementary and alternative medicine reported by persons with musculoskeletal conditions: A narrative literature synthesis
Source: PLoS One. 2018 Jul 19;13(7):e0200879. doi: 10.1371/journal.pone.0200879 (PMC6053199; doi:10.1371/journal.pone.0200879)
Supplement: S4 Appendix — (DOCX) [file pone.0200879.s004.docx]

**Appendix 4: All articles included in the narrative synthesis**

Papers from the same study are clumped together under the first article published from the study.

Abbot N, Ernst E. Patients' opinions about complementary medicine. *Forsch Komplementarmed Klass Naturheilkd.* 1997;4(3):164-168.

Afari N, Eisenberg DM, Herrell R, Goldberg J, Kleyman E, Ashton S, et al. Use of alternative treatments by Chronic Fatigue Syndrome discordant twins. *Integr Med.* 1999;2(2-3):97-103.

Al-Ajaji N, Taha A, Al-Zubier A. Prevalence of utilization of native medicine among primary care consumers. *Anglais.* 1998;19(5):551-554.

Alami S, Boutron I, Desjeux D, Hirschhorn M, Meric G, Rannou F, et al. Patients' and practitioners' views of knee osteoarthritis and its management: A qualitative interview study. *PLoS ONE.* 2011;6(5).

Al-Faris EA. The pattern of alternative medicine use among patients attending health centres in a military community in riyadh. *J Family Community Med.* 2000;7(2):17-25.

Al-Rowais N, Al-Faris E, Mohammad AG, Al-Rukban M, Abdulghani HM. Traditional healers in Riyadh region: reasons and health problems for seeking their advice. A household survey. *J Altern Complement Med.* 2010;16(2):199-204.

Anderson DL, Shane-McWhorter L, Crouch BI, Andersen SJ. Prevalence and patterns of alternative medication use in a university hospital outpatient clinic serving rheumatology and geriatric patients. *Pharmacotherapy.* 2000;20(8):958-966.

Arcury TA, Gesler WM, Cook HL. Meaning in the use of unconventional arthritis therapies. *Am J Health Promot.* 1999;14(1):7-15.

Artus M, Croft P, Lewis M. The use of CAM and conventional treatments among primary care consulters with chronic musculoskeletal pain. *BMC Fam Pract.* 2007;8:26.

Asbring P, Narvanen AL. Women's experiences of stigma in relation to chronic fatigue syndrome and fibromyalgia. *Qual Health Res.* 2002;12(2):148-160.

Astin JA, Pelletier KR, Marie A, Haskell WL. Complementary and alternative medicine use among elderly persons: one-year analysis of a Blue Shield Medicare supplement. *J Gerontol A Biol Sci Med Sci.* 2000;55(1):M4-9.

Augello JDB. *Workers with chronic pain: recovery and reintegration* [PhD]: College of Nursing, The University of Utah; 2002.

Ax S, Gregg VH, Jones D. Chronic fatigue syndrome: sufferers' evaluation of medical support. *J R Soc Med.* 1997;90(5):250-254.

Azaz-Livshits T, Muszkat M, Levy M. Use of complementary alternative medicine in patients admitted to internal medicine wards. *Int J Clin Pharm Th.* 2002;40(12):539-547.

Barnes PM, Powell-Griner E, McFann K, Nahin RL. Complementary and alternative medicine use among adults: United States, 2002. *Adv Data.* 2004(343):1-19.

- Barnes PM, Powell-Griner E, McFann K, Nahin RL. Complementary and alternative medicine use among adults: United States, 2002. *Seminars Integrative Med.* 2004;2(2):54-71.
- Bertisch SM, Wee CC, Phillips RS, McCarthy EP. Alternative mind-body therapies used by adults with medical conditions. *J Psychosom Res.* 2009;66(6):511-519.
- Bruno JJ, Ellis JJ. Herbal use among US elderly: 2002 National Health Interview Survey. *Ann Pharmacother.* 2005;39(4):643-648.
- Burke A, Upchurch DM, Dye C, Chyu L. Acupuncture use in the United States: findings from the National Health Interview Survey. *J Altern Complement Med.* 2006;12(7):639-648.
- Gardiner P, Graham R, Legedza ATR, Ahn AC, Eisenberg DM, Phillips RS. Factors associated with herbal therapy use by adults in the United States. *Alternative Therapies in Health and Medicine.* 2007;13(2):22-29.
- Kanodia AK, Legedza ATR, Davis RB, Eisenberg DM, Phillips RS. Perceived benefit of Complementary and Alternative Medicine (CAM) for back pain: a national survey. *J Am Board Fam Med.* 2010;23(3):354-362.
- Saydah SH, Eberhardt MS. Use of complementary and alternative medicine among adults with chronic diseases: United States 2002. *J Altern Complement Med.* 2006;12(8):805-812.
- Upchurch DM, Burke A, Dye C, Chyu L, Kusunoki Y, Greendale GA. A sociobehavioral model of acupuncture use, patterns, and satisfaction among women in the United States, 2002. *Womens Health Issues.* 2008;18(1):62-71.

Bernstein JH, Shuval JT. Nonconventional medicine in Israel: consultation patterns of the Israeli population and attitudes of primary care physicians. *Soc Sci Med.* 1997;44(9):1341-1348.

Bishop FL, Barlow F, Coghlan B, Lee P, Lewith GT. Patients as healthcare consumers in the public and private sectors: a qualitative study of acupuncture in the UK. *BMC Health Serv Res.* 2011;11.

Bishop FL, Yardley L, Lewith GT. Treat or treatment: a qualitative study analyzing patients' use of complementary and alternative medicine. *Am J Publich Health.* 2008;98(9):1700-1705.

Blakeley JA, Ribeiro V. A survey of self-medication practices and perceived effectiveness of glucosamine products among older adults. *Complement Ther Med.* 2002;10(3):154-160.

Borkan J, Reis S, Hermoni D, Biderman A. Talking about the pain: a patient-centered study of low back pain in primary care. *Soc Sci Med.* 1995;40(7):977-988.

Bovey M, Horner C, Shaw J, et al. Engaging in the audit of acupuncture practice. *J Altern Complement Med.* 2005;11(2):293-298.

Breuer GS, Orbach H, Elkayam O, et al. Use of complementary and alternative medicine among patients attending rheumatology clinics in Israel. *Isr Med Assoc J.* 2006;8(3):184-187.

Breuner CC, Barry PJ, Kemper KJ. Alternative medicine use by homeless youth. *Arch Pediatr Adolesc Med.* 1998;152(11):1071-1075.

Brown M, Dean S, Hay-Smith EJC, Taylor W, Baxter GD. Musculoskeletal pain and treatment choice: an exploration of illness perceptions and choices of conventional or complementary therapies. *Disabil Rehabil.* 2010;32(20):1645-1657.

Bucker B, Groenewold M, Schoefer Y, Schafer T. The use of complementary alternative medicine (CAM) in 1001 German adults: results of a population-based telephone survey. *Gesundheitswesen.* 2008;70(8-9):e29-36.

Bullock ML, Pheley AM, Kiresuk TJ, Lenz SK, Culliton PD. Characteristics and complaints of patients seeking therapy at a hospital-based alternative medicine clinic. *J Altern Complement Med.* 1997;3(1):31-37.

Calnan M, Wainwright D, O'Neill C, Winterbottom A, Watkins C. Evaluating health-care: The perspectives of sufferers with upper limb pain. *Health Expect.* 2005;8(2):149-160.

- Calnan M, Wainwright D, O'Neill C, Winterbottom A, Watkins C. Making sense of aches and pains. *Fam Pract.* 2006;23(1):91-105.

Candib LM. Making sense of my thumbs: Coming to terms with chronic illness. *Fam Syst Health.* 2004;22(2):139-151.

Carlson MJ, Krahn G. Use of complementary and alternative medicine practitioners by people with physical disabilities: estimates from a National US Survey. *Disabil Rehabil.* 2006;28(8):505-513.

Carpentier L, Prazuck T, Vincent-Ballereau F, Ouedraogo LT, Lafaix C. Choice of traditional or modern treatment in west Burkina Faso. *World Health Forum.* 1995;16(2):198-202.

Caspi O, Koithan M, Criddle MW. Alternative medicine or "alternative" patients: a qualitative study of patient-oriented decision-making processes with respect to complementary and alternative medicine. *Med Decis Making.* 2004;24(1):64-79.

Cassidy CM. Chinese medicine users in the United States. Part I: Utilization, satisfaction, medical plurality. *J Altern Complement Med.* 1998;4(1):17-27.

- Cassidy CM. Chinese medicine users in the United States - Part II: Preferred aspects of care. *J Altern Complement Med.* 1998;4(2):189-202.

Caswell AM, West J. An investigation into the factors affecting patient selection of chronic low back management methods, with particular reference to non-utilization of the complimentary therapies, in the United Kingdom. *J Back Musculoskelet Rehabil.* 2002;16(4):121-133.

Chambers S, Raine R, Rahman A, Hagley K, De Ceulaer K, Isenberg D. Factors influencing adherence to medications in a group of patients with systemic lupus erythematosus in Jamaica. *Lupus.* 2008;17(8):761-769.

Chambers SA, Raine R, Rahman A, Isenberg D. Why do patients with systemic lupus erythematosus take or fail to take their prescribed medications? A qualitative study in a UK cohort. *Rheumatology (Oxford).* 2009;48(3):266-271.

Chan H. The utilization and reasons of choice for Chinese or western medicine among rheumatoid arthritis patients in Hong Kong. *Hong Kong Nurs J.* 2002;38(3):7-17.

Chandola A, Young Y, McAlister J, Axford JS. Use of complementary therapies by patients attending musculoskeletal clinics. *J R Soc Med.* 1999;92(1):13-16.

Chandrashekara S, Anilkumar T, Jamuna S. Complementary and alternative drug therapy in arthritis. *J Assoc Physicians India.* 2002;50:225-227.

Choi Y. *Representation of and coping with osteoarthritis among older Koreans in the United States*, University of Utah; 2002.

Cushman LF, Wade C, Factor-Litvak P, Kronenberg F, Firester L. Use of complementary and alternative medicine among African-American and Hispanic women in New York City: a pilot study. *J Am Med Womens Assoc.* 1999;54(4):193-195.

DeBar LL, Vuckovic N, Schneider J, Ritenbaugh C. Use of complementary and alternative medicine for temporomandibular disorders. *J Orofac Pain.* 2003;17(3):224-236.

Dessio W, Wade C, Chao M, Kronenberg F, Cushman LF, Kalmuss D. Religion, spirituality, and healthcare choices of African-American women: Results of a national survey. *Ethn Dis.* 2004;14(2):189-197.

Dewar AL, Gregg K, White MI, Lander J. Navigating the health care system: perceptions of patients with chronic pain. *Chronic Dis Can.* 2009;29(4):162-168.

Dimmock S, Troughton PR, Bird HA. Factors predisposing to the resort of complementary therapies in patients with fibromyalgia. *Clin Rheumatol.* 1996;15(5):478-482.

Drivdahl CE, Miser WF. The use of alternative health care by a family practice population. *J Am Board Fam Pract.* 1998;11(3):193-199.

Dyck I. Managing chronic illness: an immigrant woman's acquisition and use of health care knowledge. *Am J Occup Ther.* 1992;46(8):696-705.

Eisenberg DM, Kessler RC, Van Rompay MI, Kaptchuk TJ, Wilkey SA, Appel S, et al. Perceptions about complementary therapies relative to conventional therapies among adults who use both: results from a national survey. *Ann Intern Med.* 2001;135(5):344-351.

Elder NC, Gillcrist A, Minz R. Use of alternative health care by family practice patients. *Arch Fam Med.* 1997;6(2):181-184.

Erickson BE. Radioactive pain relief: Health care strategies and risk assessment among elderly persons with arthritis at radon health mines. *J Altern Complement Med.* 2007;13(3):375-379.

Evans DD. *Characteristics and outcomes of individuals self-selecting yoga versus physical therapy for the treatment of chronic low back pain*: Nursing, The University of Tennessee Health Science Center; 2009.

- Evans DD, Carter M, Panico R, Kimble L, Morlock JT, Spears MJ. Characteristics and predictors of short-term outcomes in individuals self-selecting yoga or physical therapy for treatment of chronic low back pain. *PM&R.* 2010;2(11):1006-1015.

Figaro MK, Allegrante JP, Russo PW. Preferences for arthritis care among urban African Americans: "I don't want to be cut". *Health Psychol.* 2004;23(3):324-330.

Frank R, Stollberg G. Ayurvedic patients in Germany. *Anthropol Med.* 2002;8(3):223-244.

Gaul C, Eismann R, Schmidt T, May A, Leinisch E, Wieser T, et al. Use of complementary and alternative medicine in patients suffering from primary headache disorders. *Cephalalgia.* 2009;29(10):1069-1078.

Gaumer G. Factors associated with patient satisfaction with chiropractic care: Survey and review of the literature. *J Manipulative Physiol Ther.* 2006;29(6):455-462.

Glover-Graf N, Marini I, Baker J, Buck T. Religious and Spiritual Beliefs and Practices of Persons With Chronic Pain. *Rehabil Couns Bull.* 2007;51(1):21-23.

Goldstein MS, Glik D. Use of and satisfaction with homeopathy in a patient population. *Altern Ther Health Med.* 1998;4(2):60-65.

Gray D. The treatment strategies of arthritis sufferers. *Soc Sci Med.* 1985;21(5):507-515.

Grotenhermen F, Schnelle M. Survey on the medical use of cannabis and THC in Germany. *J Cannabis Ther.* 2003;3(2):17-41.

Hagen LE, Schneider R, Stephens D, Modrusan D, Feldman BM. Use of complementary and alternative medicine by pediatric rheumatology patients. *Arthritis & Rheumatology.* 2003;49(1):3-6.

Harrison C, Hewison J, Davies P, Pietroni P. The expectations, health beliefs and behaviour of patients seeking homoeopathic and conventional medicine. *Br Homeopath J.* 1989;78(4):210-213.

Hasan SS, Feng SR, Ahmadi K, et al. Factors influencing concomitant use of complementary and alternative medicines with warfarin. *JPPR.* 2010;40(4):294-299.

Herman CJ, Allen P, Hunt WC, Prasad A, Brady TJ. Use of complementary therapies among primary care clinic patients with arthritis. *Prev Chronic Dis.* 2004;1(4):A12.

Hestbaek L, Jorgensen A, Hartvigsen J. A description of children and adolescents in Danish chiropractic practice: results from a nationwide survey. *J Manipulative Physiol Ther.* 2009;32(8):607-615.

Hipwell AE. *Punjabi Sikh Women's Arthritis Self-management Experiences*. Coventry, Coventry University; 2009.

Ho KY, Jones L, Gan TJ. The effect of cultural background on the usage of complementary and alternative medicine for chronic pain management. *Pain Physician.* 2009;12(3):685-688.

Hori S, Mihaylov I, Vasconcelos JC, McCoubrie M. Patterns of complementary and alternative medicine use amongst outpatients in Tokyo, Japan. *BMC Complement Altern Med.* 2008;8:14.

Howell SL. Natural/alternative health care practices used by women with chronic pain: findings from a grounded theory research study. *Nurse Pract Forum.* 1994;5(2):98-106.

Hughes JG. "When I first started going I was going in on my knees, but I came out and I was skipping": exploring rheumatoid arthritis patients' perceptions of receiving treatment with acupuncture. *Complement Ther Med.* 2009;17(5-6):269-273.

Hunnicutt SE, Grady J, McNearney TA. Complementary and alternative medicine use was associated with higher perceived physical and mental functioning in early systemic sclerosis. *Explore (NY).* 2008;4(4):259-263.

Ikuyama S, Imamura-Takase E, Tokunaga S, Oribe M, Nishimura J. Sixty percent of patients with rheumatoid arthritis in Japan have used dietary supplements or health foods. *Mod Rheumatol.* 2009;19(3):253-259.

- Ikuyama S, Imamura E. Dietary supplement use in patients with rheumatoid arthritis in Japan. *Curr Rheumatol Rev.* 2009;5(4):241-245.

Ishizaki N, Yano T, Kawakita K. Public status and prevalence of acupuncture in Japan. *Evid Based Complement Alternat Med.* 2010;7(4):493-500.

James R, Fox M, Taheri G. Who goes to a natural therapist? Why? *Aust Fam Physician.* 1983;12(5):383-384, 386.

Kelner M, Wellman B. Health care and consumer choice: Medical and alternative therapies. *Soc Sci Med.* 1997;45(2):203-212.

Kestin M, Miller L, Littlejohn G, Wahlqvist M. The use of unproven remedies for rheumatoid arthritis in Australia. *Med J Aust.* 1985;143(11):516-518.

Kim C, Kwok YS. Navajo use of native healers. *Arch Intern Med.* 1998;158(20):2245-2249.

Kim HA, Seo Y-I. Use of complementary and alternative medicine by arthritis patients in a university hospital clinic serving rheumatology patients in Korea. *Rheumatol Int.* 2003;23(6):277-281.

Klingberg E, Wallerstedt SM, Torstenson T, Hawi G, Forsblad-d'Elia H. The use of complementary and alternative medicine in outpatients with inflammatory rheumatic diseases in Sweden. *Scand J Rheumatol.* 2009;38(6):472-480.

Krauss HH, Godfrey C, Kirk J, Eisenberg DM. Alternative health care: Its use by individuals with physical disabilities. *Arch Phys Med Rehab.* 1998;79(11):1440-1447.

la Cour P. Rheumatic disease and complementary-alternative treatments: a qualitative study of patient's experiences. *JCR.* 2008;14(6):332-337.

Lam TP. Strengths and weaknesses of traditional Chinese medicine and Western medicine in the eyes of some Hong Kong Chinese. *J Epidemiol Community Health.* 2001;55(10):762-765.

Lambert TD, Morrison KE, Edwards J, Clarke CE. The use of complementary and alternative medicine by patients attending a UK headache clinic. *Complement Ther Med.* 2010;18(3-4):128-134.

Lanski SL, Greenwald M, Perkins A, Simon HK. Herbal therapy use in a pediatric emergency department population: expect the unexpected. *Pediatrics.* 2003;111(5 Pt 1):981-985.

Lee M-S, Lee MS, Yang C-Y, Lee S-I, Joo M-C, Shin B-C, et al. Use of complementary and alternative medicine by rheumatoid arthritis patients in Korea. *Clin Rheumatol.* 2008;27(1):29-33.

Leiper DA, Elliott AM, Hannaford PC. Experiences and perceptions of people with headache: a qualitative study. *BMC Fam Pract.* 2006;7:27.

Lewis D, Paterson M, Beckerman S, Sandilands C. Attitudes toward integration of complementary and alternative medicine with hospital-based care. *J Altern Complement Med.* 2001;7(6):681-688.

Lim A, Cranswick N, Skull S, South M. Survey of complementary and alternative medicine use at a tertiary children's hospital. *J Paediatr Child Health.* 2005;41(8):424-427.

Lim AS, Bishop GD. The role of attitudes and beliefs in differential health care utilisation among Chinese in Singapore. *Psychol Health.* 2000;14(6):965-977.

Lim MK, Sadarangani P, Chan HL, Heng JY. Complementary and alternative medicine use in multiracial Singapore. *Complement Ther Med.* 2005;13(1):16-24.

Lloyd P, Lupton D, Wiesner D, Hasleton S. Choosing Alternative Therapy - an Exploratory-Study of Sociodemographic Characteristics and Motives of Patients Resident in Sydney. *Aust J Public Health.* 1993;17(2):135-144.

Ma GX. Between two worlds: the use of traditional and Western health services by Chinese immigrants. *J Community Health.* 1999;24(6):421-437.

MacPherson H, Scullion A, Thomas KJ, Walters S. Patient reports of adverse events associated with acupuncture treatment: A prospective national survey. *Qual Saf Health Care.* 2004;13(5):349-355.

Mak JCS, Faux S. Complementary and alternative medicine use by osteoporotic patients in Australia (CAMEO-A): a prospective study. *J Altern Complement Med.* 2010;16(5):579-584.

- Mak JCS, Faux S. Use of complementary and alternative medicine by patients with osteoporosis in Australia. *Med J Aust.* 2010;192(1):54-55.

Marsh J, Hager C, Havey T, Sprague S, Bhandari M, Bryant D. Use of alternative medicines by patients with OA that adversely interact with commonly prescribed medications. *Clin Orthop.* 2009;467(10):2705-2722.

Menniti-Ippolito F, Gargiulo L, Bologna E, Forcella E, Raschetti R. Use of unconventional medicine in Italy: a nation-wide survey. *Eur J Clin Pharmacol.* 2002;58(1):61-64.

Mitzdorf U, Beck K, Horton-Hausknecht J, Weidenhammer W, Kindermann A, Takacs M, et al. Why do patients seek treatment in hospitals of complementary medicine? *J Altern Complement Med.* 1999;5(5):463-473.

Moore J, Phipps K, Marcer D, Lewith G. Why do people seek treatment by alternative medicine? *British Medical Journal (Clinical Research Edition).* 1985;290(6461):28-29.

Murray J, Shepherd S. Alternative or additional medicine? An exploratory study in general practice. *Soc Sci Med.* 1993;37(8):983-988.

Najm W, Reinsch S, Hoehler F, Tobis J. Use of complementary and alternative medicine among the ethnic elderly. *Alternative Therapies inHealth and Medicine.* 2003;9(3):50-57.

Nayak S, Matheis RJ, Agostinelli S, Shifleft SC. The use of complementary and alternative therapies for chronic pain following spinal cord injury: a pilot survey. *J Spinal Cord Med.* 2001;24(1):54-62.

Nyiendo J, Haldeman S. A prospective study of 2,000 patients attending a chiropractic college teaching clinic. *Med Care.* 1987;25(6):516-527.

Nyman CS, Lutzen K. Caring needs of patients with rheumatoid arthritis. *Nurs Sci Q.* 1999;12(2):164-169.

Oldendick R, Coker AL, Wieland D, Raymond JI, Probst JC, Schell BJ, et al. Population-based survey of complementary and alternative medicine usage, patient satisfaction, and physician involvement. South Carolina Complementary Medicine Program Baseline Research Team. *South Med J.* 2000;93(4):375-381.

Palinkas LA, Kabongo ML, San Diego Unified Practice Research in Family Medicine Network. The use of complementary and alternative medicine by primary care patients. A SURF*NET study. *J Fam Pract.* 2000;49(12):1121-1130.

Paterson C. Patients' experiences of Western-style acupuncture: the influence of acupuncture 'dose', self-care strategies and integration. *J Health Serv Res Policy.* 2007;12 Suppl 1:S1-39-45.

Paterson C, Britten N. 'Doctors can't help much': the search for an alternative. *Br J Gen Pract.* 1999;49(445):626-629.

Peleg R, Liberman O, Press Y, Shvartzman P. Patients visiting the complementary medicine clinic for pain: A cross sectional study. *BMC Complement Altern Med.* 2011;11(36).

Peters D, Stepans MBF. Access to biofeedback therapy for women suffering from headache in rural Wyoming. *Online J Rural Nurs Health Care.* 2001;2(2).

Pitetti R, Singh S, Hornyak D, Garcia SE, Herr S. Complementary and alternative medicine use in children. *Pediatr Emerg Care.* 2001;17(3):165-169.

Qidwai W. Utilization of services of homeopathic practitioners among patients in Karachi, Pakistan. *J Ayub Med Coll Abbottabad.* 2003;15(3):33-35.

Ramos-Remus C, Gamez-Nava JI, Gonzalez-Lopez L, Skeith KJ, Perla-Navarro AV, Galvan-Villegas F, et al. Use of alternative therapies by patients with rheumatic disease in Guadalajara, Mexico: prevalence, beliefs, and expectations. *Arthritis Care Res.* 1998;11(5):411-418.

Ramos-Remus C, Watters CA, Dyke L, Suarez-Almazor ME, Russell AS. Assessment of health locus of control in the use of nonconventional remedies by patients with rheumatic diseases. *J Rheumatol.* 1999;26(11):2468-2474.

Ramsay C, Walker M, Alexander J. *Alternative Medicine in Canada: Use and Public Attitudes.* Vancouver: The Fraser Institute;1999.

Randall C, Meethan K, Randall H, Dobbs F. Nettle sting of Urtica dioica for joint pain--an exploratory study of this complementary therapy. *Complement Ther Med.* 1999;7(3):126-131.

Rao JK, Arick R, Mihaliak K, Weinberger M. Using focus groups to understand arthritis patients' perceptions about unconventional therapy. *Arthritis Care Res.* 1998;11(4):253-260.

Rao JK, Mihaliak K, Kroenke K, Bradley J, Tierney WM, Weinberger M. Use of complementary therapies for arthritis among patients of rheumatologists. *Ann Intern Med.* 1999;131(6):409-416.

- Rao JK, Kroenke K, Mihaliak KA, Grambow SC, Weinberger M. Rheumatology patients' use of complementary therapies: results from a one-year longitudinal study. *Arthritis Rheum.* 2003;49(5):619-625.

Reid J, Ewan C, Lowy E. Pilgrimage of Pain - the Illness Experiences of Women with Repetition Strain Injury and the Search for Credibility. *Soc Sci Med.* 1991;32(5):601-612.

Resch KL, Hill S, Ernst E. Use of complementary therapies by individuals with 'arthritis'. *Clin Rheumatol.* 1997;16(4):391-395.

Rose G. Why do patients with rheumatoid arthritis use complementary therapies? *Musculoskelet.* 2006;4(2):101-115.

Rossi P, Di Lorenzo G, Faroni J, Malpezzi MG, Cesarino F, Nappi G. Use of complementary and alternative medicine by patients with chronic tension-type headache: results of a headache clinic survey. *Headache.* 2006;46(4):622-631.

Rossi P, Di Lorenzo G, Malpezzi MG, Faroni J, Cesarino F, Di Lorenzo C, et al. Prevalence, pattern and predictors of use of complementary and alternative medicine (CAM) in migraine patients attending a headache clinic in Italy. *Cephalalgia.* 2005;25(7):493-506.

Rossi P, Torelli P, Di Lorenzo C, Sances G, Manzoni GC, Tassorelli C, et al. Use of complementary and alternative medicine by patients with cluster headache: results of a multi-centre headache clinic survey. *Complement Ther Med.* 2008;16(4):220-227.

Salminen E, Heikkila S, Poussa T, Lagstrom H, Saario R, Salminen S. Female patients tend to alter their diet following the diagnosis of rheumatoid arthritis and breast cancer. *Prev Med.* 2002;34(5):529-535.

Samdup DZ, Smith RG, Il Song S. The use of complementary and alternative medicine in children with chronic medical conditions. *Am J Phys Med Rehabil.* 2006;85(10):842-846.

Sanders H, Davis MF, Duncan B, Meaney FJ, Haynes J, Barton LL. Use of complementary and alternative medical therapies among children with special health care needs in southern Arizona. *Pediatrics.* 2003;111(3):584-587.

Shah SF-u-H, Mubeen SM, Mansoor S. Concepts of homeopathy among general population in Karachi, Pakistan. *J Pak Med Assoc.* 2010;60(8):667-670.

Sharples FMC, van Haselen R, Fisher P. NHS patients' perspective on complementary medicine: a survey. *Complement Ther Med.* 2003;11(4):243-248.

Shmueli A, Shuval J. Use of complementary and alternative medicine in Israel: 2000 vs. 1993. *Isr Med Assoc J.* 2004;6(1):3-8.

- Shmueli A, Shuval J. Satisfaction with Family Physicians and Specialists and the use of Complementary and Alternative Medicine in Israel. *Evid Based Complement Alternat Med.* 2006;3(2):273-278.
- Shmueli A, Igudin I, Shuval J. Change and stability: use of complementary and alternative medicine in Israel: 1993, 2000 and 2007. *Eur J Public Health.* 2011;21(2):254-259.

Shreffler-Grant J, Weinert C, Nichols E, Ide B. Complementary therapy use among older rural adults. *Public Health Nurs.* 2005;22(4):323-331.

Silverman M, Musa D, Kirsch B, Siminoff LA. Self care for chronic illness: older African Americans and whites. *J Cross Cult Gerontol.* 1999;14(2):169-189.

Simpson N, Roman K. Complementary medicine use in children: extent and reasons. A population-based study. *Br J Gen Pract.* 2001;51(472):914-916.

Singh P, Yadav RJ, Pandey A. Utilization of indigenous systems of medicine & homoeopathy in India. *Indian J Med Res.* 2005;122(2):137-142.

Singh V, Raidoo DM, Harries CS. The prevalence, patterns of usage and people's attitude towards complementary and alternative medicine (CAM) among the Indian community in Chatsworth, South Africa. *BMC Complement Altern Med.* 2004;4:3.

Sirois FM, Gick ML. An investigation of the health beliefs and motivations of complementary medicine clients. *Soc Sci Med.* 2002;55(6):1025-1037.

Skelton AM, Murphy EA, Murphy RJ, O'Dowd TC. Patients' views of low back pain and its management in general practice. *Br J Gen Pract.* 1996;46(404):153-156.

Smith JM, Sullivan SJ, Baxter GD. Massage therapy services for healthcare: A telephone focus group study of drivers for clients' continued use of services. *Complement Ther Med.* 2009;17(5-6):281-291.

Smith JM, Sullivan SJ, Baxter GD. The culture of massage therapy: valued elements and the role of comfort, contact, connection and caring. *Complement Ther Med.* 2009;17(4):181-189.

Spigelblatt L, Laineammara G, Pless IB, Guyver A. The Use of Alternative Medicine by Children. *Pediatrics.* 1994;94(6):811-814.

Sternberg SA, Chandran A, Sikka M. Alternative Therapy Use by Elderly African Americans Attending a Community Clinic. *J Am Geriatr Soc.* 2003;51(12):1768-1772.

Strauss SL. Assessing the effectiveness of acupuncture: comparison and evaluation of four retrospective surveys of the patient's opinion. *Am J Acupunct.* 1989;17(3):229-239.

Strutt R, Shaw Q, Leach J. Patients' perceptions and satisfaction with treatment in a UK osteopathic training clinic. *Manual Ther.* 2008;13(5):456-467.

Teig S, Peacock S, Stevens L, Tordoff K, Maguire E, Watson P. An audit of self acupuncture for chronic musculoskeletal pain. *Acupunct Med.* 2006;24(2):80-86.

Vincent C, Furnham A. Why do patients turn to complementary medicine? An empirical study. *Br J Clin Psychol.* 1996;35(1):37-48.

Visser GJ, Peters L, Rasker JJ. Rheumatologists and their patients who seek alternative care: an agreement to disagree. *Br J Rheumatol.* 1992;31(7):485-490.

von Peter S, Ting W, Scrivani S, Korkin E, Okvat H, Gross M, et al. Survey on the use of complementary and alternative medicine among patients with headache syndromes. *Cephalalgia.* 2002;22(5):395-400.

Wade C, Chao M, Kronenberg F, Cushman L, Kalmuss D. Medical pluralism among American women: results of a national survey. *J Womens Health (Larchmt).* 2008;17(5):829-840.

Wardwell WI. The Connecticut survey of public attitudes toward chiropractic. *J Manipulative Physiol Ther.* 1989;12(3):167-173.

Wells RE, Phillips RS, Schachter SC, McCarthy EP. Complementary and alternative medicine use among US adults with common neurological conditions. *J Neurol.* 2010;257(11):1822-1831.

- Wells RE, Bertisch SM, Buettner C, Phillips RS, McCarthy EP. Complementary and alternative medicine use among U.S. adults with migraines/severe headaches. *Headache.* 2011;51:62.

Xue CCL, Zhang AL, Lin V, Myers R, Polus B, Story DF. Acupuncture, chiropractic and osteopathy use in Australia: a national population survey. *BMC Public Health.* 2008;8:105.

Yamashita H, Tsukayama H, Sugishita C. Popularity of complementary and alternative medicine in Japan: A telephone survey. *Complement Ther Med.* 2002;10(2):84-93.

Young AE, Wasiak R, Phillips L, Gross DP. Workers' perspectives on low back pain recurrence: "It comes and goes and comes and goes, but it's always there". *Pain.* 2011;152(1):204-211.

Zaman T, Agarwal S, Handa R. Complementary and alternative medicine use in rheumatoid arthritis: an audit of patients visiting a tertiary care centre. *Natl Med J India.* 2007;20(5):236-239.

Zhang J, Verhoef MJ. Illness management strategies among Chinese immigrants living with arthritis. *Soc Sci Med.* 2002;55(10):1795-1802.
