# Supplementary material for: Justifications for using complementary and alternative medicine reported by persons with musculoskeletal conditions: A narrative literature synthesis
Source: PLoS One. 2018 Jul 19;13(7):e0200879. doi: 10.1371/journal.pone.0200879 (PMC6053199; doi:10.1371/journal.pone.0200879)
Supplement: S5 Appendix — (DOCX) [file pone.0200879.s005.docx]

**Appendix 5: All articles included in robustness testing**

Papers from the same study are clumped together under the first article published from the study.

Allen-Unhammer A, Wilson FJ, Hestbaek L. Children and adolescents presenting to chiropractors in Norway: National Health Insurance data and a detailed survey. *Chiropr Man Therap.* 2016;24:29.

Arman M, Hök J. Self-care follows from compassionate care - chronic pain patients' experience of integrative rehabilitation. *Scand J Caring Sci.* 2016;30(2):374-81.

Arya V, Thakur R, Kumar S. Consumer buying behaviour towards Ayurvedic medicines/products in Joginder Nagar- a survey. *JADR.* 2012;2(2):25-31.

Asprey A, Paterson C, White A. 'All in the same boat': a qualitative study of patients' attitudes and experiences in group acupuncture clinics. *Acupunct Med.* 2012;30(3):163-9.

Au TS, Wong MC, McMillan AS, Bridges S, McGrath C. Treatment seeking behaviour in southern Chinese elders with chronic orofacial pain: a qualitative study. *BMC Oral Health.* 2014;14:8.

Audulv A, Asplund K, Norbergh K-G. The integration of chronic illness self-management. *Qual Health Res.* 2012;22(3):332-345.

Basedow M, Runciman B, March L, Esterman A. Australians with osteoarthritis; the use of and beliefs about complementary and alternative medicines. *Complement Ther Clin Pract.* 2014;20(4):237-243.

Birhan W, Giday M, Teklehaymanot T. The contribution of traditional healers' clinics to public health care system in Addis Ababa, Ethiopia: a cross-sectional study. *J Ethnobiol Ethnomedicine.* 2011;7:39.

Bishop FL, Lewith GT. Patients' preconceptions of acupuncture: a qualitative study exploring the decisions patients make when seeking acupuncture. *BMC Complement Altern Med.* 2013;13.

Brown, T, Bonello, R, Fernandez-Caamano, R, Graham, L, Eaton, S, Green, H. Chiropractic in Australia: A Survey of the General Public. *Chiropr J Austr.* 2013;43(3): 85-93.

Boyington JE, Schoster B, Callahan LF. Comparisons of body image perceptions of a sample of Black and White women with rheumatoid arthritis and fibromyalgia in the US. *Open Rheumatol J.* 2015;9:1-7.

Bradbury KJ, Bishop FL, Yardley L, Lewith G. Patients' appraisals of public and private healthcare: a qualitative study of physiotherapy and osteopathy. *J Health Psychol.* 2013;18(10):1307-1318.

Briones-Vozmediano E, Vives-Cases C, Ronda-Perez E, Gil-Gonzalez D. Patients' and professionals' views on managing fibromyalgia. *Pain Res Manag.* 2013;18(1):19-24.

Brough N, Lindenmeyer A, Thistlethwaite J, Lewith G, Stewart-Brown S. Perspectives on the effects and mechanisms of craniosacral therapy: a qualitative study of users' views. *Eur J Integr Med.* 2015;7(2):172-183.

Brown BT, Bonello R, Fernandez-Caamano R, Eaton S, Graham PL, Green H. Consumer characteristics and perceptions of chiropractic and chiropractic services in Australia: results from a cross-sectional survey. *J Manipulative Physiol Ther.* 2014;37(4):219-229.

Burke A, Kuo T, Harvey R, Wang J. An international comparison of attitudes toward traditional and modern medicine in a Chinese and an American clinic setting. *Evid Based Complement Alternat Med.* 2011;2011:204137.

Cheshire A, Polley M, Peters D, Ridge D. Is it feasible and effective to provide osteopathy and acupuncture for patients with musculoskeletal problems in a GP setting? A service evaluation. *BMC Fam Pract.* 2011;12:49.

Cheung C. Complementary/alternative therapy use in older women with arthritis. *Res Gerontol Nurs.* 2012;5(4):275-283.

Cheung C, Geisler C, Sunneberg J. Complementary/alternative medicine use for arthritis by older women of urban-rural settings. *J Am Assoc Nurse Pract.* 2014;26(5):273-280.

- Geisler CC, Cheung CK. Complementary/alternative therapies use in older women with arthritis: information sources and factors influencing dialog with health care providers. *Geriatr Nurs.* 2015;36(1):15-20.

Cheung C, Justice C, Peden-McAlpine C. Yoga adherence in older women six months post-osteoarthritis intervention. *Glob Adv Health Med.* 2015;4(3):16-23.

Colmenares-Roa T, Huerta-Sil G, Infante-Castaneda C, Lino-Perez L, Alvarez-Hernandez E, Pelaez-Ballestas I. Doctor-Patient Relationship Between Individuals With Fibromyalgia and Rheumatologists in Public and Private Health Care in Mexico. *Qual Health Res.* 2016;26(12):1674-1688.

Combs A, Thorn E. Barriers and facilitators to yoga use in a population of individuals with self-reported chronic low back pain: a qualitative approach. *Complement Ther Clin Pract.* 2014;20(4):268-276.

Cross V, Leach CMJ, Fawkes CA, Moore AP. Patients' expectations of osteopathic care: a qualitative study. *Health Expect.* 2015;18(5):1114-1126.

Dalla Libera D, Colombo B, Pavan G, Comi G. Complementary and alternative medicine (CAM) use in an Italian cohort of pediatric headache patients: the tip of the iceberg. *Neurol Sci.* 2014;35 Suppl 1:145-148.

Dima A, Lewith GT, Little P, Moss-Morris R, Foster NE, Bishop FL. Identifying patients' beliefs about treatments for chronic low back pain in primary care: a focus group study. *Br J Gen Pract.* 2013;63(612):e490-e498.

Driban JB, Boehret SA, Balasubramanian E, Cattano NM, Glutting J, Sitler MR. Medication and supplement use for managing joint symptoms among patients with knee and hip osteoarthritis: a cross-sectional study. *BMC Musculoskelet Disord.* 2012;13.

Elder C, DeBar L, Ritenbaugh C, Vollmer W, Deyo RA, Dickerson J, et al. Acupuncture and chiropractic care: utilization and electronic medical record capture. *Am J Manag Care.* 2015;21(7):e414-421.

- Penney LS, Ritenbaugh C, Elder C, Schneider J, Deyo RA, DeBar LL. Primary care physicians, acupuncture and chiropractic clinicians, and chronic pain patients: a qualitative analysis of communication and care coordination patterns. *BMC Complement Altern Med.* 2016;16:30.

Fletcher CE, Mitchinson AR, Trumble EL, Hinshaw DB, Dusek JA. Perceptions of other integrative health therapies by Veterans with pain who are receiving massage. *J Rehabil Res Dev.* 2016;53(1):117-126.

Gaul C, Schmidt T, Czaja E, Eismann R, Zierz S. Attitudes towards complementary and alternative medicine in chronic pain syndromes: a questionnaire-based comparison between primary headache and low back pain. *BMC Complement Altern Med.* 2011;11:89.

Gong G, Li J, Li X, Mao J. Pain experiences and self-management strategies among middle-aged and older adults with arthritis. *J Clin Nurs.* 2013;22(13-14):1857-1869.

Halpin SN, Wei H, Perkins MM. Comparisons between body needle acupuncture, auricular acupuncture, and auricular magnet therapy given to veterans suffering from chronic pain. *Am Acupunct.* 2015;71:7-12.

Hennius BJ. Contemporary chiropractic practice in the UK: a field study of a chiropractor and his patients in a suburban chiropractic clinic. *Chiropr Man Therap.* 2013;21(1):25.

Hill S, Dziedzic KS, Nio Ong B. Patients' perceptions of the treatment and management of hand osteoarthritis: a focus group enquiry. *Disabil Rehabil.* 2011;33(19-20):1866-1872.

Hottenbacher L, Weisshuhn TER, Watanabe K, Seki T, Ostermann J, Witt CM. Opinions on Kampo and reasons for using it - results from a cross-sectional survey in three Japanese clinics. *BMC Complement Altern Med.* 2013;13(108).

Hsu C, Sherman KJ, Eaves ER, Turner JA, Cherkin DC, Cromp D, et al. New perspectives on patient expectations of treatment outcomes: results from qualitative interviews with patients seeking complementary and alternative medicine treatments for chronic low back pain. *BMC Complement Altern Med.* 2014;14:276.

- Eaves ER, Sherman KJ, Ritenbaugh C, Hsu C, Nichter M, Turner JA, et al. A qualitative study of changes in expectations over time among patients with chronic low back pain seeking four CAM therapies. *BMC Complement Altern Med* 2015;15(1).

Jakes D, Kirk R. How and why patients use acupuncture: an interpretive phenomenological study. *J Prim Health Care.* 2015;7(2):124-129.

Kadayat TM, Bist G, Parajuli A, Karki R, Kaundinnyayana A, Dhami N. Patterns and perception of complementary and alternative medicine use by patients in western Nepal. *J Public Health.* 2012;20(3):297-303.

Kaur K, Singh B, Kaur G. Complementary and alternative medicine usage in patients for different ailments in rural region of malwa area of punjab: a cross-sectional study. *Natl J Physiol Pharm Pharmacol.* 2016;6(5):394-398.

Kirby ER, Broom AF, Sibbritt DW, Adams J, Refshauge KM. A national cross-sectional survey of back pain care amongst Australian women aged 60-65. *Eur J Integr Med.* 2013;5(1):36-43.

- Frawley J, Sundberg T, Steel A, Sibbritt D, Broom A, Adams J. Prevalence and characteristics of women who consult with osteopathic practitioners during pregnancy; a report from the Australian Longitudinal Study on Women's Health (ALSWH). *Bodyw Mov Ther.* 2016;20(1):168-172.
- Kirby ER, Broom AF, Adams J, Sibbritt DW, Refshauge KM. A qualitative study of influences on older women's practitioner choices for back pain care. *BMC Health Serv Res.* 2014;14:131.
- Kirby E, Broom A, Sibbritt D, Refshauge K, Adams J. Suffering, recognition and reframing: Healthcare choices and plural care pathways for women with chronic back pain. *Curr Sociol.* 2015;63(5):652-668.
- Murthy V, Adams J, Broom A, Kirby E, Refshauge KM, Sibbritt D. The influence of communication and information sources upon decision-making around complementary and alternative medicine use for back pain among Australian women aged 60-65 years. *Health Soc Care Community.* 2017;25(1):114-122.
- Murthy V, Sibbritt D, Broom A, Kirby E, Frawley J, Refshauge KM, et al. Back pain sufferers' attitudes toward consultations with CAM practitioners and self- prescribed CAM products: a study of a nationally representative sample of 1310 Australian women aged 60-65 years. *Complement Ther Med.* 2015;23(6):782-788.
- Steel A, Adams J, Sibbritt D, Broom A, Gallois C, Frawley J. Determinants of women consulting with a complementary and alternative medicine practitioner for pregnancy-related health conditions. *Women Health.* 2014;54(2):127-144.

Kligler B, Buonora M, Gabison J, Jacobs E, Karasz A, McKee MD. "I felt like it was god's hands putting the needles in": a qualitative analysis of the experience of acupuncture for chronic pain in a low-income, ethnically diverse, and medically underserved patient population. *J Altern Complement Med.* 2015;21(11):713-719.

Larmer P, Kersten P, Dangan J. Patient reported benefits of hydrotherapy for arthritis. *New Zeal J Physiother.* 2014;42(2):89-94.

Leach CMJ, Hodgson L, Defever E, Ives R. Communicating risk and shared decision-making in osteopathic practice: a pilot study using focus groups to test a patient information leaflet. *Eur J Integr Med.* 2014;6(4):478-487.

Mbada CE, Adeyemi TL, Adedoyin RA, Badmus HD, Awotidebe TO, Arije OO, et al. Prevalence and modes of complementary and alternative medicine use among peasant farmers with musculoskeletal pain in a rural community in South-Western Nigeria. *BMC Complement Altern Med.* 2015;15:164.

McClymont H, Gow J, Perry C. The role of information search in seeking alternative treatment for back pain: a qualitative analysis. *Chiropr Man Therap.* 2014;22:16.

Miller JL, Teare SR, Marlett N, Shklarov S, Marshall DA. Support for living a meaningful life with osteoarthritis: a patient-to-patient research study. *Patient.* 2016;9(5):457-464.

Morfe JHD, Lim VS. Complementary and alternative medicine among Filipinos: prevalence, costs and patterns of use. *Philipp J Intern Med.* 2013;51(4).

Mothupi MC. Use of herbal medicine during pregnancy among women with access to public healthcare in Nairobi, Kenya: a cross-sectional survey. *BMC Complement Altern Med.* 2014;14:432.

Nagarajaiah BH, Kishore MS, Shashi Kumar NS, Praveen P. Prevalence and pattern of self-medication practices among population of three districts of South Karnataka. *Natl J Physiol Pharm Pharmacol.* 2016;6(4):296-300.

Nguyen D, Gavaza P, Hollon L, Nicholas R. Examination of the use of complementary and alternative medicine in Central Appalachia, USA. *Rural Remote Health.* 2014;14:2484.

Niemi M, Stahle G. The use of ayurvedic medicine in the context of health promotion--a mixed methods case study of an ayurvedic centre in Sweden. *BMC Complement Altern Med.* 2016;16:62.

Nilsen G, Anderssen N. Struggling for a normal life: work as an individual self-care management strategy among persons living with non-malignant chronic pain. *Work.* 2014;49(1):123-132.

Obalum DC, Ogo CN. Usage of complementary and alternative Medicine (CAM) among osteoarthritis patients attending an urban multi-specialist hospital in Lagos, Nigeria. *Niger Postgrad Med J.* 2011;18(1):44-47.

Onofri E, Mercuri M, Tadonkeng MC. Use of phytotherapy in relation to conventional medicines: Habits and beliefs. *Gazz Med Ital Arch Sci Med..* 2016;175(1-2):27-33.

Orrock PJ. The patient experience of osteopathic healthcare. *Manual Ther.* 2016;22:131-137.

Osborne A, Blake C, Meredith D, McNamara J, Phelan J, Cunningham C. The lived experience of low back pain among Irish farmers: case studies. *J Agromedicine.* 2014;19(2):181-191.

Park J, Hirz E, Manotas K, Hooyman N. Nonpharmacological pain management by ethnically diverse older adults with chronic pain: barriers and facilitators. *J Gerontol Soc Work.* 2013;56(6):487-509.

Park J, Lavin R, Couturier B. Choice of nonpharmacological pain therapies by ethnically diverse older adults. *Pain Manag.* 2014;4(6):389-406.

Parsons S, Harding G, Breen A, Foster N, Pincus T, Vogel S, et al. Will shared decision making between patients with chronic musculoskeletal pain and physiotherapists, osteopaths and chiropractors improve patient care? *Fam Pract.* 2012;29(2):203-212.

Pedersen IK. 'It can do no harm': Body maintenance and modification in alternative medicine acknowledged as a non risk health regimen. *Soc Sci Med.* 2013;90:56-62.

Pedersen W, Sandberg S. The medicalisation of revolt: a sociological analysis of medical cannabis users. *Sociol Health Illn.* 2013;35(1):17-32.

Pollard-Smith T, Thomson OP. Professional ballet dancers' experience of injury and osteopathic treatment in the UK: a qualitative study. *J Bodyw Mov Ther.* 2016.

Pugh JD, Williams AM. Feldenkrais method empowers adults with chronic back pain. *Holist Nurs Pract.* 2014;28(3):171-183.

Rajendran D, Bright P, Bettles S, Carnes D, Mullinger B. What puts the adverse in 'adverse events'? Patients' perceptions of post-treatment experiences in osteopathy--a qualitative study using focus groups. *Manual Ther.* 2012;17(4):305-311.

Ramamoorthy A, Jeevakarunyam SJ, Janardhanan S, Jeddy N, Vasan SA, Raja A, et al. Survey on utility of yoga as an alternative therapy for occupational hazards among dental practioners. *J Nat Sci Biol Med.* 2015;6(1):149-152.

Rutledge DN, Cantero PJ, Ruiz JE. Chronic pain management strategies used by low-income overweight Latinos. *Chronic Illn*. 2013;9(2):133-44.

Sadiq S, Kaur S, Khajuria V, Gupta S, Sharma A. Complementary and alternative medicine use in medical OPD patients of rheumatoid arthritis in a tertiary care hospital. *Natl J Physiol Pharm Pharmacol.* 2016;6(4):305-309.

Sadr S, Pourkiani-Allah-Abad N, Stuber KJ. The treatment experience of patients with low back pain during pregnancy and their chiropractors: a qualitative study. *Chiropr Man Therap.* 2012;20(1):32.

Selten EM, Vriezekolk JE, Geenen R, van der Laan WH, van der Meulen-Dilling RG, Nijhof MW, et al. Reasons for treatment choices in knee and hip osteoarthritis: a qualitative study. *Arthritis Care Res.* 2016;68(9):1260-7.

Seo HJ, Sung YK, Choi CB, Lee EB, Cheong C, Kim SY, et al. Prevalence and factors affecting glucosamine use in Korea: a survey-based study. *Rheumatol Int.* 2013;33(6):1627-1631.

Serbic D, Pincus T. Chasing the ghosts: The impact of diagnostic labelling on self-management and pain-related guilt in chronic low back pain patients. *J Pain Manag.* 2013;6(1):25-35.

Silvanathan S, Low BS. Current public awareness on the safety of traditional and complementary medicines (T&CM) in Malaysia. *Eur J Integr Med.* 2015;7(2):184-189.

Singh JA. Facilitators and barriers to adherence to urate-lowering therapy in African-Americans with gout: a qualitative study. *Arthritis Res Ther.* 2014;16(2).

Sirois FM, Salamonsen A, Kristoffersen AE. Reasons for continuing use of Complementary and Alternative Medicine (CAM) in students: a consumer commitment model. *BMC Complement Altern Med.* 2016;16.

Son HM, Kim DH, Kim E, Jung SY, Kim AR, Kim TH. A qualitative study of the experiences of patients with knee osteoarthritis undergoing moxibustion. *Acupunct Med.* 2013;31(1):39-44.

Soner BC, Sahin AS, Sahin TK. A survey of Turkish hospital patients' use of herbal medicine. *Eur J Integr Med.* 2013;5(6):547-552.

Stomski NJ, Mackintosh SF, Stanley M. The experience of acupuncture care from the perspective of people with chronic low back pain: a grounded theory study. *Acupunct Med.* 2014;32(4):333-339.

Stoneman P, Sturgis P, Allum N. Understanding support for complementary and alternative medicine in general populations: use and perceived efficacy. *Health.* 2013;17(5):512-529.

Sviland R, Martinsen K, Raheim M. To be held and to hold one's own: narratives of embodied transformation in the treatment of long lasting musculoskeletal problems. *Med Health Care Philos.* 2014;17(4):609-624.

*Tait EM, Laditka JN, Laditka SB, Nies MA, Racine EF, Tsulukidze MM. Reasons why older Americans use complementary and alternative medicine: costly or ineffective conventional medicine and recommendations from health care providers, family, and friends. *Educ Gerontol.* 2013;39(9):684-700.

- Burke A, Nahin RL, Stussman BJ. Limited health knowledge as a reason for non-use of four common complementary health practices. *PLoS ONE.* 2015;10(6):e0129336.
- Johnson PJ, Kozhimannil KB, Jou J, Ghildayal N, Rockwood TH. Complementary and alternative medicine use among women of reproductive age in the United States. *Womens Health Issues.* 2016;26(1):40-47.
- Upchurch DM, Rainisch BW. A sociobehavioral wellness model of acupuncture use in the United States, 2007. *J Altern Complement Med.* 2014;20(1):32-39.

Tippens KM, Chao MT, Connelly E, Locke A. Patient perspectives on care received at community acupuncture clinics: a qualitative thematic analysis. *BMC Complement Altern Med.* 2013;13.

Tsui T, Boon H, Boecker A, Kachan N, Krahn M. Understanding the role of scientific evidence in consumer evaluation of natural health products for osteoarthritis an application of the means end chain approach. *BMC Complement Altern Med.* 2012;12.

Ulusoy H, Gucer TK, Aksu M, Arslan S, Habiboglu A, Akgol G, et al. The use of complementary and alternative medicine in Turkish patients with rheumatic diseases. *Turk J Rheumatol.* 2012;27(1):31-37.

Ward L, Treharne GJ, Stebbings S. The suitability of yoga as a potential therapeutic intervention for rheumatoid arthritis: a focus group approach. *Musculoskeletal Care*. 2011;9(4):211-21.

Weeks WB, Goertz CM, Meeker WC, Marchiori DM. Public perceptions of doctors of chiropractic: results of a national survey and examination of variation according to respondents' likelihood to use chiropractic, experience with chiropractic, and chiropractic supply in local health care markets. *J Manipulative Physiol Ther*. 2015;38(8):533-44.

- Weeks WB, Goertz CM, Meeker WC, Marchiori DM. Characteristics of US Adults Who Have Positive and Negative Perceptions of Doctors of Chiropractic and Chiropractic Care. *J Manipulative Physiol Ther*. 2016;39(3):150-7.

Williamson J, Ramirez R, Wingfield T. Health, healthcare access, and use of traditional versus modern medicine in remote Peruvian Amazon communities: a descriptive study of knowledge, attitudes, and practices. *Am J Trop Med Hyg.* 2015;92(4):857-864.

Wilson IM, Doherty L, McKeown L. Perceptions of playing-related musculoskeletal disorders (PRMDs) in Irish traditional musicians: a focus group study. *Work.* 2014;49(4):679-688.

Winit-Watjana W, Bello N, Baqir W, Worsley A. Prevalence and perceived outcomes of complementary and alternative medicine use in hospitalized British patients. *Arch Pharm Pract.* 2013;3(4):265-273.

Zhang Y, Leach MJ, Hall H, Sundberg T, Ward L, Sibbritt D, et al. Differences between male and female consumers of complementary and alternative medicine in a national US population: a secondary analysis of 2012 NIHS data. *Evid Based Complement Alternat Med.* 2015;2015:413173.

* Papers from a study included in the original narrative synthesis (Wells RE, Phillips RS, Schachter SC, McCarthy EP. Complementary and alternative medicine use among US adults with common neurological conditions. *J Neurol.* 2010;257(11):1822-1831.)
